# Lysyl-tRNA synthetase as a drug target in malaria and cryptosporidiosis

**DOI:** 10.1073/pnas.1814685116

**Published:** 2019-03-20

**Authors:** Beatriz Baragaña, Barbara Forte, Ryan Choi, Stephen Nakazawa Hewitt, Juan A. Bueren-Calabuig, João Pedro Pisco, Caroline Peet, David M. Dranow, David A. Robinson, Chimed Jansen, Neil R. Norcross, Sumiti Vinayak, Mark Anderson, Carrie F. Brooks, Caitlin A. Cooper, Sebastian Damerow, Michael Delves, Karen Dowers, James Duffy, Thomas E. Edwards, Irene Hallyburton, Benjamin G. Horst, Matthew A. Hulverson, Liam Ferguson, María Belén Jiménez-Díaz, Rajiv S. Jumani, Donald D. Lorimer, Melissa S. Love, Steven Maher, Holly Matthews, Case W. McNamara, Peter Miller, Sandra O’Neill, Kayode K. Ojo, Maria Osuna-Cabello, Erika Pinto, John Post, Jennifer Riley, Matthias Rottmann, Laura M. Sanz, Paul Scullion, Arvind Sharma, Sharon M. Shepherd, Yoko Shishikura, Frederick R. C. Simeons, Erin E. Stebbins, Laste Stojanovski, Ursula Straschil, Fabio K. Tamaki, Jevgenia Tamjar, Leah S. Torrie, Amélie Vantaux, Benoît Witkowski, Sergio Wittlin, Manickam Yogavel, Fabio Zuccotto, Iñigo Angulo-Barturen, Robert Sinden, Jake Baum, Francisco-Javier Gamo, Pascal Mäser, Dennis E. Kyle, Elizabeth A. Winzeler, Peter J. Myler, Paul G. Wyatt, David Floyd, David Matthews, Amit Sharma, Boris Striepen, Christopher D. Huston, David W. Gray, Alan H. Fairlamb, Andrei V. Pisliakov, Chris Walpole, Kevin D. Read, Wesley C. Van Voorhis, Ian H. Gilbert

**Affiliations:** ^a^Wellcome Centre for Anti-Infectives Research, Drug Discovery Unit, Division of Biological Chemistry and Drug Discovery, University of Dundee, DD1 5EH Dundee, United Kingdom;; ^b^Seattle Structural Genomics Center for Infectious Disease, Seattle, WA 98109;; ^c^Division of Allergy and Infectious Diseases, University of Washington, Seattle, WA 98109;; ^d^Center for Emerging and Re-emerging Infectious Diseases, University of Washington, Seattle, WA 98109;; ^e^Beryllium Discovery Corp., Bainbridge Island, WA 98110;; ^f^Center for Tropical and Emerging Global Diseases, University of Georgia, Athens, GA 30602;; ^g^Department of Life Sciences, Imperial College, South Kensington, SW7 2AZ London, United Kingdom;; ^h^Medicines for Malaria Venture, 1215 Geneva 15, Switzerland;; ^i^The Art of Discovery, 48160 Derio, Bizkaia, Basque Country, Spain;; ^j^Department of Medicine, University of Vermont, Burlington, VT 05405;; ^k^Biology Department, Calibr at Scripps Research, La Jolla, CA 92037;; ^l^Department of Medical Parasitology and Infection Biology, Swiss Tropical and Public Health Institute, CH-4002 Basel, Switzerland;; ^m^Universität Basel, CH-4003 Basel, Switzerland;; ^n^Diseases of the Developing World, Global Health, GlaxoSmithKline, 28760 Tres Cantos, Madrid, Spain;; ^o^Structural Parasitology Group, International Centre for Genetic Engineering and Biotechnology, 110067 New Delhi, India;; ^p^Malaria Molecular Epidemiology Unit, Institut Pasteur du Cambodge, 12 201 Phnom Penh, Cambodia;; ^q^Skaggs School of Pharmaceutical Sciences, University of California, San Diego, La Jolla, CA 92093;; ^r^Department of Pediatrics, School of Medicine, University of California, San Diego, La Jolla, CA 92093;; ^s^Center for Global Infectious Disease Research, Seattle Children’s Research Institute, Seattle, WA 98109;; ^t^Department of Global Health, University of Washington, Seattle, WA 98195;; ^u^Department of Biomedical Informatics and Medical Education, University of Washington, Seattle, WA 98195;; ^v^Structural Genomics Consortium, University of Toronto, Toronto, ON M5G 1L7, Canada;; ^w^Department of Pathobiology, School of Veterinary Medicine, University of Pennsylvania, Philadelphia, PA 19104;; ^x^Computational Biology, School of Life Sciences, University of Dundee, DD1 5EH Dundee, United Kingdom;; ^y^Physics, School of Science and Engineering, University of Dundee, Dundee DD1 4HN, United Kingdom;; ^z^Structural Genomics Consortium, Research Institute of the McGill University Health Centre, Montreal, QC H4A 3J1, Canada

**Keywords:** malaria, cryptosporidiosis, tRNA synthetase

## Abstract

Malaria and cryptosporidiosis, caused by apicomplexan parasites, remain major drivers of global child mortality. New drugs for the treatment of malaria and cryptosporidiosis, in particular, are of high priority; however, there are few chemically validated targets. The natural product cladosporin is active against blood- and liver-stage *Plasmodium falciparum* and *Cryptosporidium parvum* in cell-culture studies. Target deconvolution in *P. falciparum* has shown that cladosporin inhibits lysyl-tRNA synthetase (*Pf*KRS1). Here, we report the identification of a series of selective inhibitors of apicomplexan KRSs. Following a biochemical screen, a small-molecule hit was identified and then optimized by using a structure-based approach, supported by structures of both *Pf*KRS1 and *C. parvum* KRS (*Cp*KRS). In vivo proof of concept was established in an SCID mouse model of malaria, after oral administration (ED_90_ = 1.5 mg/kg, once a day for 4 d). Furthermore, we successfully identified an opportunity for pathogen hopping based on the structural homology between *Pf*KRS1 and *Cp*KRS. This series of compounds inhibit *Cp*KRS and *C. parvum* and *Cryptosporidium hominis* in culture, and our lead compound shows oral efficacy in two cryptosporidiosis mouse models. X-ray crystallography and molecular dynamics simulations have provided a model to rationalize the selectivity of our compounds for *Pf*KRS1 and *Cp*KRS vs. (human) *Hs*KRS. Our work validates apicomplexan KRSs as promising targets for the development of drugs for malaria and cryptosporidiosis.

## Significance

Malaria and cryptosporidiosis are major burdens to both global health and economic development in many countries. Malaria caused >400,000 deaths in 2017, and cryptosporidiosis is estimated to cause >200,000 deaths a year. The spread of drug resistance is a growing concern for malaria treatment, and there is no effective treatment for malnourished or immunocompromised children infected with *Cryptosporidium*. New treatments with novel mechanisms of action are needed for both diseases. We present a selective inhibitor of both *Plasmodium* and *Cryptosporidium* lysyl-tRNA synthetase capable of clearing parasites from mouse models of malaria and cryptosporidiosis infection. This provides very strong validation of lysyl-tRNA synthetase as a drug target in these organisms and a lead for further drug discovery.

Malaria is caused by *Plasmodium spp.*; the most significant species from a disease perspective are *Plasmodium falciparum* and *Plasmodium vivax*. In 2017, there were estimated to be 219 million clinical cases of malaria and 435,000 deaths from the disease (1, 2). There is an urgent need for new drugs for malaria to deal with the constant threat of drug resistance and to provide new drugs for chemoprotection, prevention of transmission, and treatment of relapsing (vivax) malaria (2). In humans, cryptosporidiosis is predominantly caused by *Cryptosporidium hominis* and *Cryptosporidium parvum*. The recent Global Enteric Multicenter Study has highlighted cryptosporidiosis as a leading cause of moderate-to-severe diarrheal diseases in infants. The association of cryptosporidiosis with death was the highest for any pathogen in 6- to 18-mo-old children with moderate-to-severe diarrhea (3–5). Cryptosporidiosis is estimated to lead to >200,000 deaths a year and is also associated with malnutrition, stunted growth, and cognitive-development problems in children (6). The currently approved drug nitazoxanide has poor efficacy, particularly in the case of immune-compromised patients and malnourished children, where there is no effective treatment (7, 8).

In the last decade, *Plasmodium* aminoacyl-tRNA synthetases have received increased attention as new targets for antimalarial drug discovery (9). Aminoacyl-tRNA synthetases catalyze aminoacylation of tRNAs with their cognate amino acids in two stages (10). First, the amino acid is activated by ATP to yield the AMP-activated amino acid, with loss of pyrophosphate, followed by transfer of the amino acid onto the tRNA. By way of example, a series of novel antimalarial bicyclic azetidines, identified by phenotypic screening, were found to inhibit cytosolic *Plasmodium* phenylalanyl-tRNA synthetase (11). These compounds showed activity across multiple life stages of the parasite and in vivo efficacy in a malaria mouse model. Malaria parasite genomes encode two different lysyl-tRNA synthetases (KRSs) that play a role in translation in either the cytoplasm (*Pf*KRS1) or in the apicoplast (*Pf*KRS2) (9, 12, 13), while *Cryptosporidium* parasites and humans encode one copy. Human KRS (*Hs*KRS) is found in both the cytosol and mitochondrion and has additional roles within human cells (14).

**Fig. 1.**
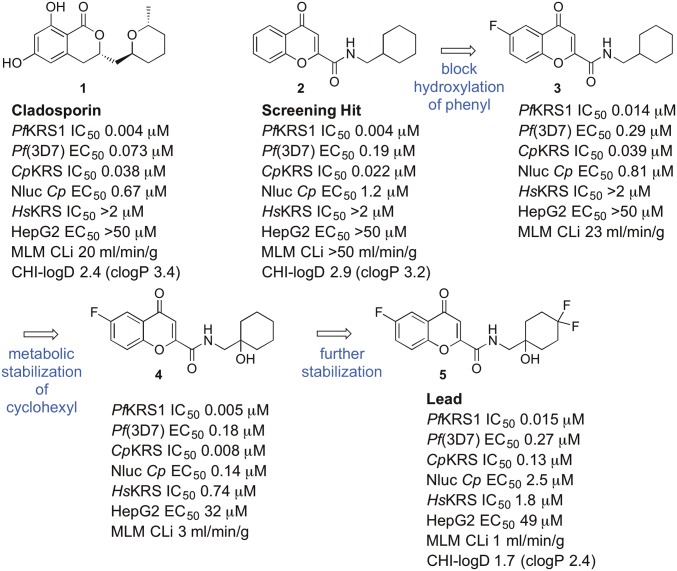
The structure of cladosporin **1**, evolution of the lead **5** from the screening hit **2**. Enzyme data were obtained with the Kinase-Glo assay. MLM, mouse liver microsomes.

Hoepfner et al. (15) discovered that the fungal secondary metabolite cladosporin **1** (Fig. 1) was a nanomolar inhibitor of parasite growth in both blood and liver stages of *Plasmodium*. They demonstrated that cladosporin inhibited cytosolic *Pf*KRS1 with >100-fold selectivity compared with *Hs*KRS. Unfortunately, cladosporin is not amenable to development as a drug lead itself because of high metabolic instability (see data we generated in Fig. 1), which would mean that it would not be significantly orally bioavailable. In this work, we report the discovery and optimization of drug-like inhibitors against *Pf*KRS1 and *Cp*KRS, which showed oral activity in mouse models of malaria and cryptosporidiosis.

## Results and Discussion

### Characterization of KRS Enzymes.

We produced recombinant *Pf*KRS1 (77–583 and 80–583), *Cp*KRS (46-end), and *Hs*KRS (full-length) proteins and developed biochemical assays based on the luciferase ATP consumption assay (Kinase-Glo; Promega) (16), which was suitable for the high-throughput screening, and the pyrophosphate generation assay (EnzChek) (17) format for kinetic characterization of the enzymes. The activities of recombinant enzymes were analyzed by monitoring only the first stage of the aminoacylation reaction. This reaction is suitable for high-throughput screening campaigns and makes the reaction more amenable for steady-state kinetic studies. By using the EnzChek assay, which monitors the production of pyrophosphate (when coupled with pyrophosphatase), *K*_m_ values were obtained for *Pf*KRS1, *Cp*KRS, and *Hs*KRS for ATP and l-lysine (Table 1 and *SI Appendix*, Fig. S1 and Table S1). The *K*_m_ values for the human enzyme are significantly smaller than for the parasite enzymes. This may be due to structural differences (see below) between the active sites of the parasite and human enzymes. Even so, the *K*_m_ values for ATP and l-lysine obtained are comparable with KRSs from other species (https://www.brenda-enzymes.org/) (18).

**Table 1.** Kinetic parameters for KRS determined by using EnzCheck

Enzyme	*K*_m_ ATP, µM*	Hill_ATP_	*K*_m_ Lys, µM^†^	Hill_l-Lys_
*Pf*KRS1	68 ± 3	—	413 ± 37	0.89 ± 0.04
*Cp*KRS	346 ± 128	0.71 ± 0.09	1,045 ± 640	0.49 ± 0.06
*Hs*KRS	2.22 ± 0.44	—	1.92 ± 0.37	—

* Determined in the presence of saturating concentration of the cosubstrate l-lysine, 5 mM l-lysine for *Pf* and *Cp* and 0.075 mM for *Hs*KRS.

^†^ Determined in the presence of saturating concentration of the cosubstrate ATP, 0.5 mM ATP for *Pf*, 2 mM for *Cp*, and 0.1 mM for *Hs*KRS.

### Hit and Lead Discovery.

By using the luciferase ATP consumption (Kinase-Glo) assay platform with sub-*K*_m_ substrate concentrations (thus biasing the assay toward identifying ATP-competitive inhibitors), the GlaxoSmithKline malaria actives set of ∼13,000 compounds (the Tres Cantos Antimalarial Set) (19) was screened against recombinant *Pf*KRS1, leading to the discovery of a *Pf*KRS1 inhibitor, compound **2** (Fig. 1). Compound **2** displayed similar levels of inhibition of *Pf*KRS1 and growth of *P. falciparum* to cladosporin **1**. Compound **2** suffered from high metabolic instability (*C*_Li_ > 50 mL⋅min^−1^⋅g^−1^ in mouse liver microsomes); however, in contrast to cladosporin **1**, it was chemically tractable.

**Fig. 2.**
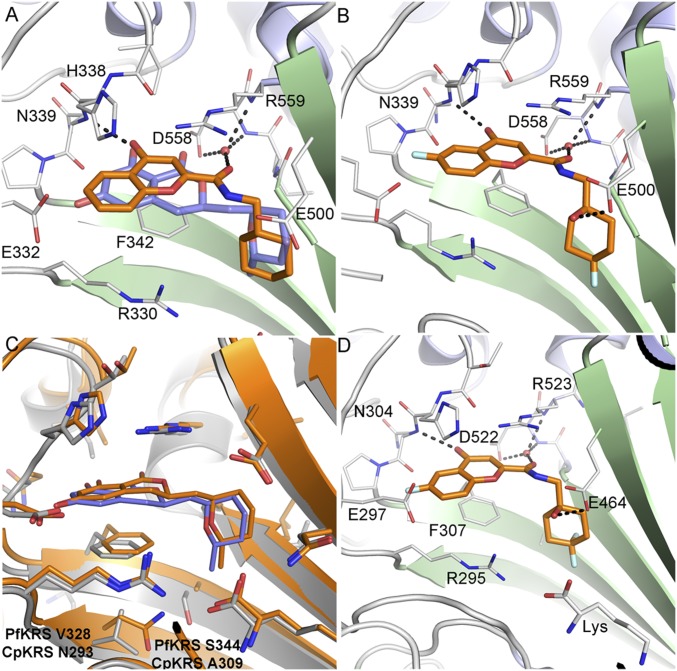
Binding modes of ligands bound to *Pf*KRS1 and *Cp*KRS. (*A*) *Pf*KRS1:Lys:**2** showing the binding mode of **2** (C atoms, gold) bound to the ATP site of *Pf*KRS1 (PDB ID code 6AGT) superimposed upon *Pf*KRS1:Lys:cladosporin (cladosporin C atoms, slate; PDB ID code 4PG3). (*B*) *Pf*KRS1:**5** showing binding mode of **5** bound to *Pf*KRS1 (PDB ID code 6HCU). (*C*) Overlay of *Cp*KRS:Lys:cladosporin (C atoms, gold; PDB ID code 5ELO) compared with *Pf*KRS1:Lys:cladosporin (C atoms, gray; PDB ID code 4PG3). Nonconserved residues within the ligand binding site are labeled. (*D*) *Cp*KRS:Lys:**5** showing binding mode of **5** (C atoms, gold) in complex with *Cp*KRS:Lys (C atoms, gray; PDB ID code 6HCW). H-bonds are shown as dashed lines, and key residues are labeled for clarity.

In the published structure of cladosporin bound to *Pf*KRS1, cladosporin binds within the ATP binding pocket (20). The isocoumarin moiety occupies the same space as the adenine ring of ATP, and the pyran system occupies the same position as the ribose ring of ATP. The two phenolic hydroxy groups of the isocoumarin ring form hydrogen bonds with the side chain of E332 and the backbone NH of N339, while the carbonyl interacts with a highly coordinated conserved water molecule (20). Screening hit **2** was cocrystallized with *Pf*KRS1 and also binds in the ATP binding pocket (Fig. 2*A*), in a similar fashion to cladosporin, although the bicyclic core is rotated 30° with respect to cladosporin. The chromone core stacks between the side chain of F342 on one face and the side chains of H338 and R559 on the other. The ring carbonyl forms an H-bond to the backbone NH of N339, mimicking the N1 of adenine and the O1 OH of cladosporin. The amide carbonyl H-bonds to a highly conserved water molecule coordinated by the side chain of D558 and the backbone NHs of D558 and R559. The cyclohexyl moiety projects into a pocket formed by the side chains of R330, F342, and S344 and the backbone of L555 and G556. This pocket is completed by the substrate lysine and is similar to that occupied by the pyran ring of cladosporin, except that the cyclohexyl ring probes deeper into the pocket (Fig. 2*A*).

Metabolite-identification studies suggested that hydroxylation occurred in both the phenyl ring of the chromone and the cyclohexyl ring. By preparing several potential metabolites, we identified the major site of hydroxylation as carbon-6 at the phenyl ring. Addition of a fluorine in the phenyl ring at C-6 blocked hydroxylation at the phenyl ring of the chromone (compound **3**; Fig. 1), and introduction of a hydroxyl at the bridgehead of the cyclohexyl substituent was tolerated without loss of potency, while reducing lipophilicity and intrinsic clearance (compound **4**). The cocomplex of **4** bound to *Pf*KRS1 showed that the ligand retained the binding mode of **2**, conserving key H-bonds from the core scaffold to the protein. The addition of the 6F atom did not afford new interactions with the protein or ordered solvent. The bridgehead hydroxyl was close to the side chain of E500, forming a weak interaction (3.4 Å) and interacting with the ordered water network (*SI Appendix*, Fig. S7). Addition of fluorines on the 4-position of the cyclohexyl ring in **5** was tolerated and led to excellent metabolic stability, both in mouse and human liver microsomes. (Fig. 1*B* and Table 2). The complex of **5** bound to *Pf*KRS1 showed that the addition of the difluoro moiety on the cyclohexyl ring had minimal effect upon the position of the ligand within the binding site with respect to **4**, and there was no evidence of protein rearrangement. In this complex, all polar interactions were retained, although the H-bond between the bridge-head OH and the side chain of Glu-500 had shortened to 3.0 Å (Fig. 2*B*).

**Table 2.** In vitro activity and DMPK profile of lead compound 5

Assay	Data
*P. falciparum* 3D7 EC_50_	0.27 μM
*P. vivax* liver schizonts/hypnozoites EC_50_ (prophylactic mode)	0.95 μM/>10 μM
*P. berghei* liver schizonts EC_50_	0.9 μM
*P. falciparum* stage V gametocytes EC_50_	9.9 μM
*P. falciparum* male/female gamete formation EC_50_	>1 μM
FaSSIF solubility	255 μM
Microsomal stability C_Lint_	1 (mouse), <0.5 (human) mL⋅min^−1^⋅g^−1^
Hepatocyte stability C_Lint_	0.5 (mouse), <0.5 (human) mL⋅min^−1^⋅g^−1^
CYP inhibition (CYP1A2, 2D6, 2C9, 2C19, 3A4) IC_50_	>10 μM
Mouse PK intravenously (dose, Clb, AUC, *T*_1/2_, Vdss)	3 mg/kg, 3.4 mL⋅min^−1^⋅kg^−1^, 890 μg⋅min/mL, 2.5 h, 1 L/kg
Mouse PK orally (dose, *T*_max_, *C*_max_, AUC_0–1440_, F%)	10 mg/kg, 2 h, 5.4 μg/mL, 1,300–3,000 μg⋅min/mL, 100%

Enzymatic studies of the inhibition of *Pf*KRS1 by compound **5** were performed by using the pyrophosphate generation (EnzChek) platform. In the presence of saturating concentrations of both substrates, an IC_50_ of 210 nM was obtained (*SI Appendix*, Fig. S2*A*). To study the mechanism of inhibition by compound **5**, single-inhibition measurements were performed at a fixed saturating concentration of one substrate and fixed variable concentrations of the second substrate. Under our experimental conditions, results showed a linear competitive inhibition vs. ATP with a *K*_*i*_ of 32 nM and a linear uncompetitive inhibition vs. l*-lysine* with a *K*_i_ of 212 nM (*SI Appendix*, Fig. S2 *B* and *C* and Table S4). These results indicate that compound **5** competes with ATP for the same binding site and only binds in the presence of l-lysine, also suggesting a sequential ordered kinetic mechanism where l-lysine is the first substrate to bind. The results also show that, in the presence of high concentrations of ATP, the binding affinity of compound **5** is reduced, whereas in the presence of high concentrations of l-lysine, it is increased. Because the mode of inhibition studies are performed at saturating concentration of the cosubstrate, this leads to a lower, more potent *K*_*i*_ against ATP (l-lysine is saturating) and a higher, less potent *K*_*i*_ against l-lysine (ATP is saturating). It is noteworthy that the selectivity ratio for *Hs*KRS/*Pf*KRS1 (120-fold in Kinase-Glo) is similar to the 180-fold cellular selectivity observed between *P. falciparum* parasites and human HepG2 cells.

**Fig. 3.**
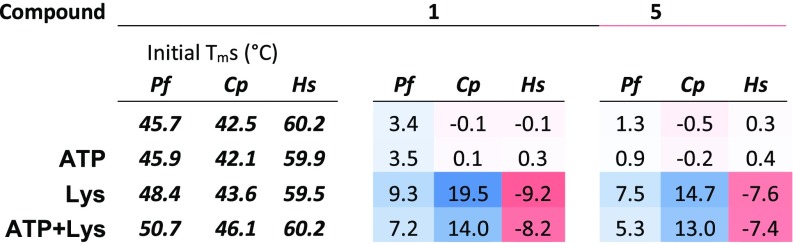
Heatmap showing effects of compounds **1** and **5** on the melting temperature (Δ*T*_*m*_) of KRS enzymes.

It was reported that cladosporin binds to *Pf*KRS1 in a cooperative manner with l-lysine, leading to significant thermal stabilization (increased melting temperature, *T*_*m*_) (21). Notably, this stabilization effect was not observed in the human counterpart. To determine whether the chromone series retained a similar stabilization effect, KRS enzymes were incubated with inhibitor and substrates (ATP and l-lysine) in various combinations and gradually heated for observable shifts in *T*_*m*_. For both *Pf*KRS and *Cp*KRS enzymes, a marked shift (>2 °C) was observed when l-lysine was present, suggesting an analogous codependent binding mode (Fig. 3). This agrees with the results of the study of the mechanism of inhibition of *Pf*KRS1 by compound **5**, in which there is a higher *K*_*i*_ determined for this compound in the presence of l-lysine. In contrast, the *Hs*KRS exhibited a reduction in *T*_m_ in the presence of inhibitor and l-lysine.

Lead compound **5** showed good systemic exposure after oral dosing with excellent oral bioavailability (*F* = 100%) and moderate half-life (*T*_1/2_ = 2.5 h) (Table 2). A preliminary selectivity study in a 44 receptor/enzyme panel showed no activity at a concentration of 10 μM. The compound did not show inhibition of a range of cytochrome P450 enzymes and did not inhibit hERG (EC_50_ > 100 μM). While the compound has a good profile in in vitro assays, compound **5** showed toxicity in mice at higher doses (50 mg/kg orally) and was itself not suitable for further progression. It is likely that the toxicity at higher doses is due to inhibition of mammalian KRS. Indeed, at a dose of 50 mg/kg, the blood concentration of compound **5** in mice reached the EC_50_ for HepG2 cells. Nonetheless, this compound is a drug-like tool compound to explore KRSs as drug targets.

### Profile in Malaria.

Compound **5** was active against both *Pf*KRS1 (IC_50_ = 0.015 μM) and whole-cell bloodstream *P. falciparum* 3D7 (EC_50_ = 0.27 μM) and was selective compared with both the *Hs*KRS (IC_50_ = 1.8 μM) and HepG2 cells (EC_50_ = 49 μM). The drop-off from enzyme to cell is probably due to multiple factors—but the two most likely are that high levels of enzyme inhibition may be required for a phenotypic response and the 1,000-fold increase in the concentration of ATP between the enzyme assay and within the parasite cell (22), given that ATP competes for binding with our inhibitors. The activity of **5** against parasites resistant to chloroquine *Pf*(K1) (EC_50_ = 0.51 μM) or atovaquone *Pf*(TM90C2B) (EC_50_ = 0.52 μM) is similar to the drug-sensitive strain *Pf*(NF54) (EC_50_ = 0.39 μM). We investigated the activity against different life-cycle stages of malaria (Table 2). The lead compound **5** showed comparable activity in liver schizonts (*P. vivax* liver schizont EC_50_ = 0.95 μM) to asexual blood stages. The in vitro parasite reduction ratio (PRR) assay (23) identified **5** as a compound with a slow rate of killing, displaying an overall biological profile similar to other *Plasmodium* protein-synthesis inhibitors acting on cytosolic targets and to atovaquone (24) (*SI Appendix*, Fig. S3).

**Fig. 4.**
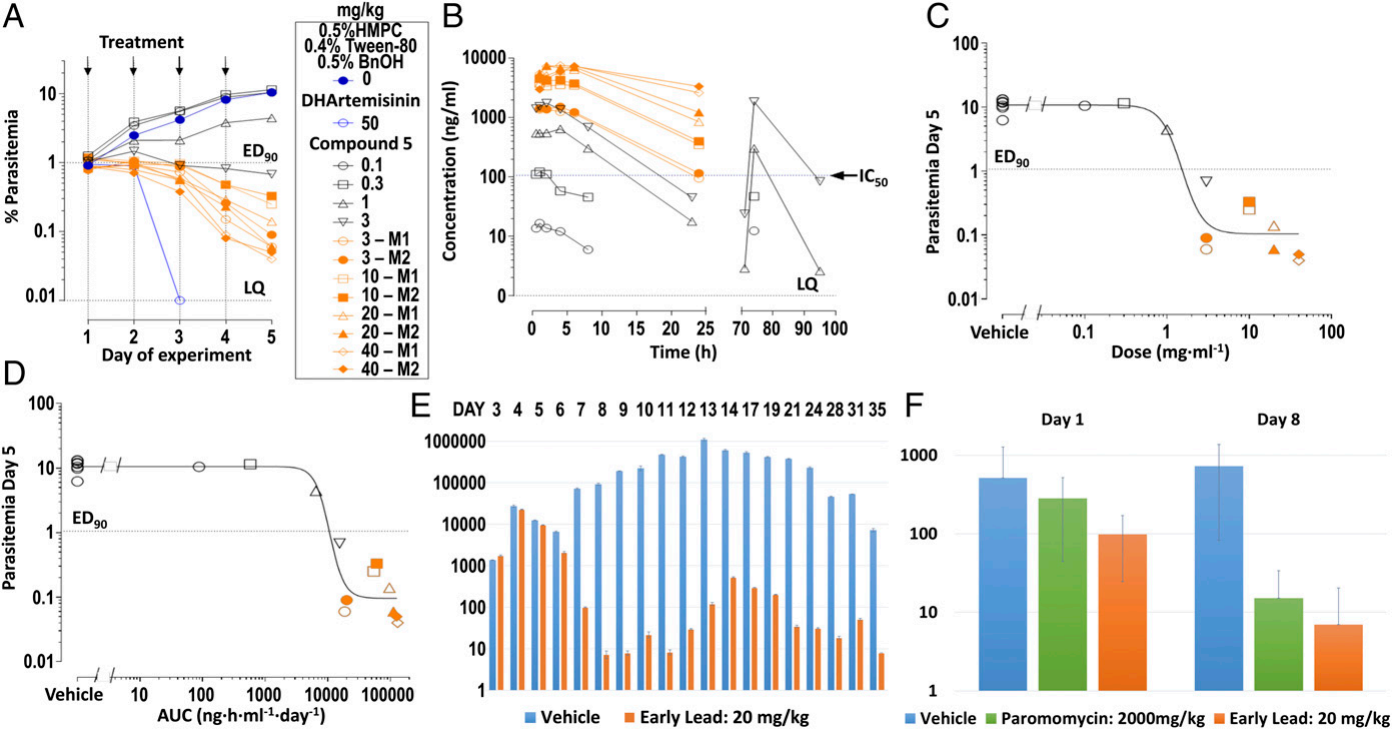
In vivo efficacy of compound **5** in mouse models of malaria and cryptosporidiosis infections. (*A*) The in vivo efficacy data for compound **5** in *P. falciparum*-infected SCID mice. (*B*) The levels of compound **5** in blood of the mice during the malaria efficacy experiment, 24 h after the first oral dose and 24 h after the administration of the last dose on day 4. The symbols represent the same individuals depicted in plot *A*. (*C*) Determination of daily dose to reduce *P. falciparum* parasitemia by 90% at day 5 of the experiment. (*D*) Determination of daily exposure in blood to reduce *P. falciparum* parasitemia by 90% at day 5 of the experiment. (*E*) Efficacy of compound **5** in the Nluc-cryptosporidiosis INF-γ–knockout mouse model when dosed orally at a concentration of 20 mg/kg once a day for 7 d. Orange bars show fecal parasite levels for mice treated with compound **5** and blue bars for the vehicle-treated control mice. At day 8, there is a 10,000-fold reduction in parasite levels compared with control. *n* = 4 mice per group. (*F*) Efficacy of compound **5** in the cryptosporidiosis NOD SCID gamma mouse model when dosed orally at a concentration of 20 mg/kg once a day for 7 d. Orange bars represent fecal parasite levels for mice treated with compound **5**, green bars mice treated with paromomycin (2,000 mg/kg), and blue bars for vehicle-treated control mice. At day 8, there is a 100-fold reduction in parasite levels compared with control.

The biological and pharmacokinetic profile was sufficient to justify a rodent efficacy study. Compound **5** was evaluated in vivo against *P. falciparum* parasites grown in the peripheral blood of NODscidIL2Rγnull mice (SCID), engrafted with human erythrocytes (25). Three days after infection, mice were dosed orally once a day for 4 d with **5**, at concentrations up to 40 mg/kg (Fig. 4*A*). From dose–response studies, a daily oral dose of ED_90_ = 1.5 mg⋅kg^−1^ (1.0–2.3 mg⋅kg^−1^) (Fig. 4*C*), or its equivalent estimated daily exposure in blood AUC_ED90_ = 11,000 ng⋅h⋅ml^−1^⋅d^−1^ (6,900–14,000 ng⋅h⋅ml^−1^⋅d^−1^) (Fig. 4*D*), reduced parasitemia by 90% at day 5 of the study. The rate of parasite clearance in vivo is consistent with the PRR data in vitro.

### Pathogen Hopping: Cryptosporidiosis.

There is a high level of sequence identity within the active-site region of *Pf*KRS1 and *Cp*KRS (96% identity) and an overall sequence identity of 47.7% and similarity of 64.6% across the entire protein. Furthermore, structurally, the active sites are very similar. Therefore, we tested cladosporin, the screening hit **2**, and the lead compound **5** in a cellular assay against *C. parvum*. The three compounds showed inhibition of parasite growth with EC_50_ of 0.7, 1.2, and 2.5 µM, respectively. Our lead **5** is similarly active against a small panel of isolates: *C. hominis* (TU502) (EC_50_ = 6.0 µM) and the *C. parvum* Iowa strain (EC_50_ = 1.3 µM). In time-kill curve studies conducted by using *C. parvum* in the HCT-8 cell-culture system (26), both cladosporin and compound **5** eliminated parasites at an exponential rate, consistent with other protein synthesis inhibitors studied to date (*SI Appendix*, Fig. S4). Subsequently, a crystal structure was determined of *Cp*KRS in complex with cladosporin and l-lysine [Protein Data Bank (PDB) ID code 5ELO] (Fig. 2*C*), showing retention of the ligand binding mode compared with the *Pf*KRS1 structure and the high level of sequence conservation within the active site, with only two sequence differences at the base of the ligand binding site (*Pf*KRS1 V328 S344 and *Cp*KRS N293 A309). Further *Cp*KRS:ligand structures were obtained for several compounds within the chromone series, including *Cp*KRS:**5** (Fig. 2*D*), showing **5** to bind in an identical manner to *Cp*KRS as to *Pf*KRS1. In cryptosporidiosis, the parasite is found predominantly in the epithelial cells (enterocytes) in the gastrointestinal tract (8), although it is thought that there may also be some parasites present in the biliary tract. Therefore, it is likely that a compound used for treating cryptosporidiosis would need to have a good exposure in the gastrointestinal tract and possibly also some systemic exposure (27). After oral dosing, compound **5** was completely bioavailable. However, some compound was present in mouse stools (17% of oral dose), suggesting that some biliary excretion had occurred. This raises the possibility of deliberately utilizing enterohepatic recirculation to maintain both gastrointestinal and systemic exposure. Compound **5** showed in vivo efficacy in two different *Cryptosporidium* mouse models, the NOD SCID gamma and INF-γ–knockout mouse models.

INF-γ–knockout mice (28, 29) were infected orally with Nluc-expressing transgenic *C. parvum* oocysts. Treatment started upon patency 4 d postinfection (p.i.), and mice were treated orally once a day for 7 d. Infection was monitored daily by luciferase measurements in pooled feces of the entire cage. Mice were followed for 3 wk after completion of drug treatment. Compound **5**, when dosed orally at 20 mg/kg once a day for 7 d, reduced parasite shedding below detection level, and this reduction was sustained for 3 wk after treatment had stopped (Fig. 4*E*). NOD SCID gamma mice were infected with *C. parvum* oocysts (26). Treatment started 7 d p.i., and mice were treated orally once a day for 7 d. The study was run with four mice per cage; infection was monitored by quantitative PCR on day eight for individual mice, and data are shown as oocysts per milligram of feces. Compound **5** dosed orally at a concentration of 20 mg/kg once a day for 7 d showed 96% reduction of parasite shedding comparable to paromomycin (Fig. 4*F*).

### Molecular Basis of *Pf*KRS1 Inhibitor Selectivity.

Molecular dynamics (MD) simulations were successful in reproducing the binding pose and interactions of compound **5** observed in the co-crystal structure of *Pf*KRS1 (*SI Appendix*, Fig. S5*A*). In addition to those interactions, the inhibitor was found to be stabilized by hydrophobic contacts established between the cyclohexyl moiety and the bound substrate l-lysine. Simulations performed in the absence of l-lysine showed a notable destabilization of compound **5**, suggesting a key role of l-lysine in the binding of *Pf*KRS1 inhibitors (*SI Appendix*, Fig. S5*B*). This was confirmed by both the structural information and the thermal shift assays (Figs. 2 and 3).

**Fig. 5.**
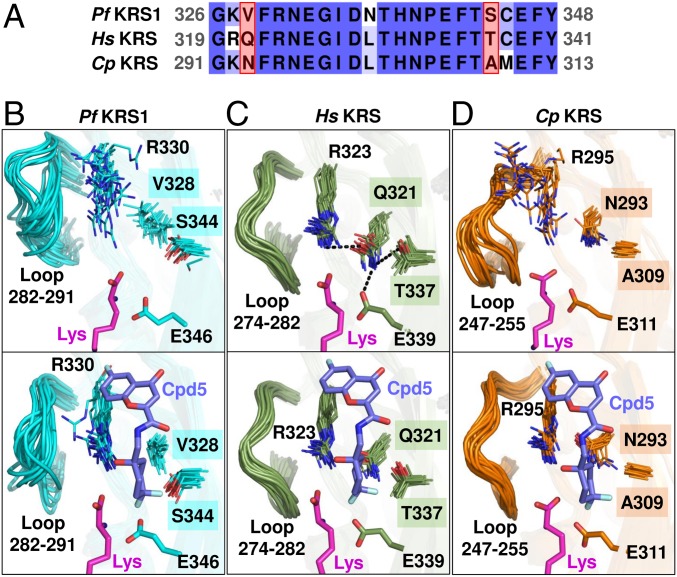
Differential binding mode of compound **5** to *Pf*KRS1, *Hs*KRS, and *Cp*KRS. (*A*) Sequence alignment of the active sites of *Pf*KRS1, *Hs*KRS, and *Cp*KRS. Two nonconserved residues suggested to be responsible for the selectivity of compound **5** are highlighted in red boxes. (*B*–*D*) Multiframe representations of the active site from the MD simulations of the active sites of *Pf*KRS1 (*B*) (cyan), *Hs*KRS (*C*) (green), and *Cp*KRS (*D*) (orange) in the absence (*Upper*) and presence (*Lower*) of compound **5**. Lysine is shown in magenta, compound **5** is shown in purple, and nonconserved residue labels are highlighted in boxes.

MD simulations of *Hs*KRS predicted a similar binding mode of compound **5** to that in *Pf*KRS1, and the per-residue contributions to the ligand binding energy also showed very similar results (*SI Appendix*, Fig. S5 *C* and *E*). However, despite the overall high sequence and structural similarity between *Pf*KRS1 and *Hs*KRS, two nonconserved residues were present within the active site: V328 and S344 in *Pf*KRS1 correspond to bulkier residues Q321 and T337 in *Hs*KRS (Fig. 5 *A*–*C*). To investigate whether this subtle difference might have an impact on the binding process of compound **5**, both enzymes were also simulated in the absence of inhibitor (*apo* systems), with the main focus placed on the conformational features of the binding pocket. In apo-*Pf*KRS1, due to the smaller size of V328 and S344 side chains, the ligand binding pocket remained accessible to compound **5** (Fig. 5*B*). Conversely, in apo-*Hs*KRS, the binding site remained partially inaccessible due the extended side chain of Q321, which formed a hydrogen-bond network with R323, T337, and E339 (Fig. 5*C* and *SI Appendix*, Fig. S6*A*). Hence, a disruption of the hydrogen-bond interactions within the active site of *Hs*KRS is required to enable the binding of the inhibitor.

The comparison of *apo* and *holo Pf*KRS1 and *Hs*KRS systems also showed significant differences in the dynamics of residues neighboring the active site. In apo-*Pf*KRS1, R330 was highly flexible and was stabilized only after binding of the inhibitor. Similar behavior was observed for the loop 282–291, which was highly disordered in the absence of the ligand, but became ordered upon ligand binding (Fig. 5*B* and *SI Appendix*, Fig. S6*C*). This was corroborated by the marked positive shift in *Pf*KRS1’s *T*_*m*_ in the presence of inhibitor and l-lysine compared with the *apo* state (Fig. 3). On the other hand, in *Hs*KRS, the equivalent R323 and loop 274–282 remained highly stable, regardless of the presence of the ligand (Fig. 5*C* and *SI Appendix*, Fig. S6 *B* and *C*). Such ligand-induced stabilization observed for the mobile loop and residues near the *Pf*KRS1 active site could potentially account for a more favorable binding of compound **5** to *Pf*KRS1 with respect to *Hs*KRS. The *Cp*KRS system exhibited behavior similar to *Pf*KRS1: an accessible binding site and a high degree of flexibility of the loop and R295 in the apo-state and dramatic stabilization upon ligand binding, which, again, was supported by the large observed shift in *T*_*m*_ from the *apo* state to the l-lysine plus inhibitor state (Figs. 3 and 5*D* and *SI Appendix*, Fig. S6 *B* and *C*). This provided a rationale for the compound **5** affinity toward *Cp*KRS.

The results of MD simulations suggested that the parasite KRS selectivity vs. *Hs*KRS observed for compound **5** was due to a combination of two factors: (*i*) a more favorable (i.e., more accessible) configuration of the binding site in the parasite enzyme, and (*ii*) a higher degree of stabilization for the *Pf*KRS1 and *Cp*KRS residues upon ligand binding. Our findings are in good agreement with previous experimental and structural studies that reported an increased flexibility of *Pf*KRS1 over *Hs*KRS and suggested that the active-site loops in aminoacyl-RNA transferases are likely to have a critical role in specific ligand recognition (21, 30, 31). It is likely that full understanding of the observed selectivity can only be obtained by reproducing the entire process of ligand binding to KRS.

In conclusion, identification of the molecular targets of phenotypic hits and subsequent target-based approaches to these targets is a promising way to develop new antiinfective agents. *Pf*KRS1 was shown to be the target of the natural product cladosporin, which was found to be active against *P. falciparum* in cell culture. Cladosporin itself is not suitable for progression to animal studies, as it is not metabolically stable or orally bioavailable. Given that cladosporin has a complex synthesis with low overall yield (eight steps with an overall yield of 8%) (32), chemical modification to improve the metabolic stability looked challenging, and a long synthesis means that the cost of goods would likely fall outside the Target Product Profiles for malaria and cryptosporidiosis. Therefore, we carried out a small-molecule screen against *Pf*KRS1 to find an alternate chemotype for optimization. Following optimization of a hit molecule (**2**), we identified a metabolically stable and orally bioavailable compound (**5**) which inhibited *Pf*KRS1 selectively. A low oral dose (1.5 mg/kg once a day for 4 d) of our KRS inhibitor, compound **5**, reduced parasitemia by 90% in the malaria SCID mouse model. These results represent an in vivo validation of *Pf*KRS1 as a promising antimalarial target for drug development. We have also successfully undertaken pathogen-hopping (33), demonstrating that this compound series inhibits *Cp*KRS and the growth of *C. parvum* in vitro. Moreover, compound **5** showed a reduction of parasite burden by two orders of magnitude when dosed orally for 7 d in two different mouse models of cryptosporidiosis. There are very few validated targets for cryptosporidiosis, and here we chemically validated in vitro and in vivo *Cp*KRS as a target for drug development.

We have carried out MD simulations to understand the observed selectivity of compound **5** and other analogs for *Pf*KRS1 and *Cp*KRS vs. *Hs*KRS. MD simulations suggest that the selectivity observed for compound **5** is due to a combination of a more favorable configuration of the binding site and a higher degree of stabilization upon ligand binding in the parasite enzymes. This work represents a strong validation of lysyl tRNA synthetase as a drug target in both malaria and cryptosporidiosis in animal models. We have identified a valuable tool compound, although further optimization is required in terms of both potency and selectivity to obtain a preclinical candidate.

## Materials and Methods

Full details are in *SI Appendix*. This includes the following information: (*i*) the chemical synthesis of compounds described in the paper; (*ii*) the methods for protein expression and purification, kinetic characterization of enzymes, screening of library, and mode of inhibition studies; (*iii*) the methods for parasite assays using the different life-cycle stages of *P. falciparum* and different species of *Cryptosporidium*; (*iv*) methods for in vitro drug metabolism and pharmacokinetic assay; (*v*) methods for in vivo pharmacokinetics and efficacy studies; (*vi*) details for molecular modeling and dynamics simulations; (*vii*) details of X-ray crystallography; (*viii*) ethical use of animals; and (*ix*) detailed author contributions. Ethical approval for rodent experiments was given by the University of Vermont Institutional Animal Care and Use Committee, The Art of Discovery Institutional Animal Care and Use Committee (TAD-IACUC), Veterinäramt Basel Stadt, the University of Dundee “Welfare and Ethical Use of Animals Committee,” and the University of Georgia Animal Care and Use Committee. Human biological samples were sourced ethically and used under informed consent.

## Supplementary Material

Supplementary File
