# Supplementary material for: Lysyl-tRNA synthetase as a drug target in malaria and cryptosporidiosis
Source: Proc Natl Acad Sci U S A. 2019 Mar 20;116(14):7015–20. doi: 10.1073/pnas.1814685116 (PMC6452685; doi:10.1073/pnas.1814685116)
Supplement: Supplementary File [file pnas.1814685116.sapp.pdf]

SUPPLEMENTARY INFORMATION

Lysyl-tRNA synthetase as a drug target in malaria and cryptosporidiosis

Beatriz Baragaña,[a] Barbara Forte,[a] Ryan Choi,[b,c,d] Stephen Nakazawa Hewitt,[b,c,d] Juan A. Bueren-Calabuig,[a] João Pedro Pisco,[a] Caroline Peet,[a] David M. Dranow,[b,e] David A. Robinson,[a] Chimed Jansen,[a] Neil R. Norcross,[a] Sumiti Vinayak,[f,#] Mark Anderson,[a] Carrie F. Brooks,[f] Caitlin A. Cooper,[f] Sebastian Damerow,[a] Michael Delves,[g] Karen Dowers,[a] James Duffy,[gh] Thomas E. Edwards, [b,e] Irene Hallyburton,[a] Benjamin G. Horst,[b,c,d] Matthew A. Hulverson,[c,d] Liam Ferguson,[a] María Belén Jiménez-Díaz,[i] Rajiv S. Jumani,[j] Donald D. Lorimer,[b,e] Melissa S. Love,[k] Steven Maher,[f] Holly Matthews,[g] Case W. McNamara,[k] Peter Miller,[j] Sandra O'Neill,[a] Kayode K. Ojo,[c,d] Maria Osuna-Cabello,[a] Erika Pinto,[a] John Post,[a] Jennifer Riley,[a] Matthias Rottmann,[l,m] Laura M. Sanz,[n] Paul Scullion,[a] Arvind Sharma,[o] Sharon M. Shepherd,[a] Yoko Shishikura,[a] Frederick R. C. Simeons,[a] Erin E. Stebbins,[j] Laste Stojanovski,[a] Ursula Straschil,[g] Fabio K. Tamaki,[a] Jevgenia Tamjar,[a] Leah S. Torrie,[a] Amélie Vantaux,[p] Benoît Witkowski,[p] Sergio Wittlin,[l,m] Manickam Yogavel,[o] Fabio Zuccotto,[a] Iñigo Angulo-Barturen,[i] Robert Sinden,[g] Jake Baum,[g] Francisco-Javier Gamo,[n] Pascal Mäser,[l,m] Dennis E. Kyle,[f] Elizabeth A. Winzeler,[q,r] Peter J. Myler,[b,s,t,u] Paul G. Wyatt,[a] David Floyd,[v] David Matthews,[v] Amit Sharma,[o] Boris Striepen,[f,w] Christopher D. Huston,[j] David W. Gray,[a] Andrei V. Pisliakov,[x,y] Alan H. Fairlamb,[a] Chris Walpole,[z] Kevin D. Read,[a] Wesley C. Van Voorhis,[b.c,d] Ian H. Gilbert[a,*]

- Author for correspondence. i.h.gilbert@dundee.ac.uk

a. Wellcome Centre for Anti-Infectives Research, Drug Discovery Unit, Division of Biological Chemistry and Drug Discovery, University of Dundee, Dundee, DD1 5EH, UK. **b**. Seattle Structural Genomics Center for Infectious Disease (SSGCID), Seattle, Washington 98109, USA. **c**. Division of Allergy and Infectious Diseases, Center for Emerging and Re-emerging Infectious Diseases, University of Washington, Seattle, WA 98109, USA. **d**. Center for Emerging and Re-emerging Infectious Diseases, University of Washington, Seattle, WA 98109, USA. **e**. Beryllium Discovery Corp., 7869 Day Road West, Bainbridge Island, Washington 98110, USA. **f**. Center for Tropical and Emerging Global Diseases, University of Georgia, Athens, GA 30602, USA**. g.** Department of Life Sciences, Sir Alexander Fleming Building, Imperial College, South Kensington, London, SW7 2AZ, UK. **h**. Medicines for Malaria Venture, 20 Route de Pré-Bois, 1215 Geneva 15, Switzerland. **i**. The Art of Discovery (TAD), Biscay Science and Technology Park, Astondo Bidea, BIC Bizkaia building, nº 612, Derio 48160, Bizkaia, Basque Country, Spain. **j**. Department of Medicine, University of Vermont, Stafford Hall, 95 Carrigan Drive, Burlington, Vermont 05405, USA. **k**. Calibr at Scripps Research Institute, 11119 N Torrey Pines

Road, Suite 100, La Jolla, CA, 92037, USA. **l**. Swiss Tropical and Public Health Institute, Socinstrasse 57, P.O. Box, CH-4002 Basel, Switzerland. **m**. Universität Basel, Petersplatz 1, CH-4003 Basel, Switzerland. **n**. Diseases of the Developing World, Global Health, GlaxoSmithKline, 28760 Tres Cantos, Madrid, Spain. **o**. Structural Parasitology Group, International Centre for Genetic Engineering and Biotechnology, Aruna Asaf Ali Road, New Delhi 110067, India. **p**. Malaria Molecular Epidemiology Unit, Institut Pasteur du Cambodge, 5 Boulevard Monivong-PO Box 983, 12 201 Phnom Penh, Cambodia. **q**. Department of Pediatrics and Skaggs School of Pharmaceutical Sciences, University of California, San Diego, School of Medicine, 9500 Gilman Drive MC 0760, La Jolla, CA 92093, USA. **r.** Department of Pediatrics, School of Medicine, University of California, San Diego, La Jolla, CA 92093, USA. **s**. Center for Infectious Disease Research (formerly Seattle Biomedical Research Institute), 307 Westlake Avenue North, Suite 500, Seattle, Washington 98109, USA. t. Departments of Global Health and Biomedical Informatics and Medical Education, University of Washington, Seattle, Washington 98195, USA. **u.** Department of Biomedical Informatics and Medical Education, University of Washington, Seattle, WA 98195, USA. **v**. Structural Genomics Consortium, University of Toronto, MaRS South Tower, Suite 705, 101 College Street, Toronto, Ontario, M5G 1L7, Canada. **w**. Department of Pathobiology, School of Veterinary Medicine, University of Pennsylvania, Philadelphia, PA 19104, USA. **x**. Computational Biology, School of Life Sciences, University of Dundee, DD1 5EH, UK. **y**. Physics, School of Science and Engineering, University of Dundee, DD1 4HN, UK. **z**. Structural Genomics Consortium, Research Institute of the McGill University Health Centre, 1001 Boul Décarie, Site Glen Block E, ES1.1614, Montreal, QC H4A 3J1, Canada

# Current address: Department of Pathobiology, College of Veterinary Medicine, University of Illinois at Urbana Champaign, Urbana IL 61802, USA

SUPPLEMENTARY INFORMATION

Contents

LIST OF FIGURES 5

LIST OF TABLES 5

1. CHEMISTRY 6

2. BIOCHEMISTRY 9

2.1 PROTEIN EXPRESSION AND PURIFICATION METHOD FOR RECOMBINANT KRS (UW) 9
2.2 PROTEIN EXPRESSION AND PURIFICATION METHOD FOR RECOMBINANT *PF*KRS1 (DUNDEE) 9
2.3 PROTEIN EXPRESSION AND PURIFICATION METHOD FOR RECOMBINANT *CP*KRS (DUNDEE) 10
2.4 PROTEIN EXPRESSION AND PURIFICATION METHOD FOR RECOMBINANT *HS*KRS (DUNDEE) 10
2.5 *PF*KRS1, *CP*KRS AND *HS*KRS PYROPHOSPHATE GENERATION ENZCHEK™ KINETIC MEASUREMENTS 11
2.6 *PF*KRS1 HIT DISCOVERY USING LUCIFERASE ATP CONSUMPTION (KINASE GLO®) (UW) 13
2.7 *PF*KRS1, *CP*KRS AND *HS*KRS SAR ASSAY USING LUCIFERASE ATP CONSUMPTION (KINASE GLO®) 13
2.8 *PF*KRS1 MODE OF INHIBITION STUDIES BY COMPOUND 5 USING PYROPHOSPHATE GENERATION (ENZCHEK™) 13
2.9 RECOMBINANT KRS THERMAL SHIFT ASSAYS (UW) 16

3. CELL BIOLOGY 16

3.1 ASSAY METHODOLOGY FOR *PLASMODIUM FALCIPARUM* (3D7) (DUNDEE). 16
3.2 CYTOTOXICITY STUDIES (DUNDEE) 17
3.3 *IN VITRO* CELL ASSAY DATA ANALYSIS (DUNDEE) 17
3.4 ASSAY METHODOLOGY FOR DRUG RESISTANT *PLASMODIUM FALCIPARUM* (K1 AND TM90C2B) (SWISSTPH). 17
3.5 *IN VITRO* PARASITE REDUCTION RATIO (PRR) ASSAY WITH *PLASMODIUM FALCIPARUM (GSK)* 17
3.6 INHIBITION OF NANOLUCIFERASE EXPRESSING *C. PARVUM* GROWTH IN HCT-8 CELLS. (UW) 18
3.7 ASSAY METHOD FOR MEASUREMENT OF *IN VITRO* INHIBITION OF *CRYPTOSPORIDIUM*. (VERMONT) 18
3.8 *IN VITRO* CRYPTOSPORIDIUM TIME-KILL CURVE ASSAY (VERMONT) 19
3.9 *PLASMODIUM VIVAX* LIVER SCHIZONTS/ HYPNOZOITES (UNIVERSITY OF GEORGIA) 20
3.10 *PLASMODIUM BERGHEI* LIVER SCHIZONTS (UCSD) 20
3.11 *PLASMODIUM FALCIPARUM* STAGE V GAMETOCYTES (UCSD) 20
3.12 *PLASMODIUM FALCIPARUM* GAMETE FORMATION (IMPERIAL) 21
3.13 ACTIVITY AGAINST *CRYPTOSPORIDIUM HOMINIS* (CALIBR) 21

4. DMPK (DUNDEE) 22

4.1 AQUEOUS SOLUBILITY 22
4.2 FASSIF SOLUBILITY 22
4.3 CHI LOGD 23
4.4 INTRINSIC CLEARANCE (CLI) EXPERIMENTS 23
4.5 HEPATOCYTE STABILITY 24
4.7 FLUORESCENCE-BASED CYP INHIBITION USING RECOMBINANTLY EXPRESSED CYP BACTOSOMES 25
4. 8 HUMAN ETHER-À-GO-GO RELATED GENE (HERG) K^+ ASSAY (OUTSOURCED) 25

5. *IN VIVO* PHARMACOKINETIC AND EFFICACY STUDIES 26

5.1 IN VIVO PHARMACOKINETICS (DUNDEE) 26
5.2 IN VIVO ANTIMALARIAL EFFICACY STUDIES IN *P. FALCIPARUM* (SWISSTPH) 26

5.3 In vivo **ANTIMALARIAL EFFICACY STUDIES IN *P. falciparum* (TAD)** 26
5.4 MEASUREMENT OF ANTI-CRYPTOSPORIDIUM *IN VIVO* EFFICACY IN IFN-Γ-KNOCKOUT MICE (GEORGIA) 28
5.5 MEASUREMENT OF ANTI-CRYPTOSPORIDIUM *IN VIVO* EFFICACY IN NOD SCID GAMMA MICE (VERMONT) 28

6. MODELLING AND MOLECULAR DYNAMICS SIMULATIONS 29

6.1 INITIAL MODELS 29
6.2 SYSTEM PREPARATION. 29
6.3 MD SIMULATION PROTOCOL. 29
6.4 MD ANALYSIS. 29

7. X-RAY CRYSTALLOGRAPHY 33

7.1 *Pf*KRS1 COMPLEXES (NEW DELHI, DUNDEE) 33
***7.2 Cp*KRS COMPLEXES (BERYLLIUM DISCOVERY, SSGCID, DUNDEE)** 34

8. ETHICAL USE OF ANIMALS AND HUMAN TISSUE 36

AUTHOR CONTRIBUTIONS 38

REFERENCES 42

List of Figures

Figure S1. Steady-state kinetics of *Pf*, *Cp* and *Hs*KRS (EnzChek). S12
Figure S2. Steady-state kinetics of *Pf*KRS1 inhibition by compound **5**. S15
Figure S3. The rate of kill of *P. falciparum* by compound **5**. S18
Figure S4. The rate of kill of *C. parvum* by cladosporin (A) and compound **5** (B). S19
Figure S5: Modelling of *Pf*KRS1, *Hs*KRS and *Cp*KRS in complex with compound **5**. S30-31
Figure S6: Differential flexibility of residues near the active site in *Pf*KRS1, *Hs*KRS and *Cp*KRS. S32
Figure S7: Binding mode of compound **5** with *Pf*KRS1. S36

List of Tables

Table S1. Steady-state kinetic parameters for *Pf*, *Cp* and *Hs*KRS (EnzChek). S12
Table S2. Steady-state kinetic parameters for *Pf*KRS1. S14
Table S3. Thermal shift data for compounds *with PfKRS1, CpKRS and HsKRS.* S16
Table S4. Summary of MD simulations. S33
Table S5. Data measurement and refinement statistics for KRS complexes. S35

1. Chemistry

Normal phase TLCs were carried out on pre-coated silica plates (Kieselgel 60 F_{254}, BDH) with visualisation *via* U.V. light (UV254/365 nm) and/or ninhydrin solution. Flash chromatography was performed using Combiflash Companion Rf (commercially available from Teledyne ISCO) and prepacked silica gel columns purchased from Teledyne ISCO. Mass-directed preparative HPLC separations were performed using a Waters HPLC (2545 binary gradient pumps, 515 HPLC make up pump, 2767 sample manager) connected to a Waters 2998 photodiode array and a Waters 3100 mass detector. Preparative HPLC separations were performed with a Gilson HPLC (321 pumps, 819 injection module, 215 liquid handler/injector) connected to a Gilson 155 UV/vis detector. On both instruments, HPLC chromatographic separations were conducted using Waters XBridge C18 columns, 19 x 100 mm, 5 um particle size; using 0.1% ammonia in water (solvent A) and acetonitrile (solvent B) or 0.1% formic acid in water (solvent A) and acetonitrile (solvent B) as mobile phase. ^{1}H NMR spectra were recorded on a Bruker Avance DPX 500 spectrometer (^{1}H at 500.1 MHz, ^{13}C at 125 MHz ^{19}F at 470.5 MHz), or a Bruker Avance DPX 300 (^{1}H at 300 MHz). Chemical shifts (δ) are expressed in ppm recorded using the residual solvent as the internal reference in all cases. Signal splitting patterns are described as singlet (s), doublet (d), triplet (t), quartet (q), multiplet (m), broad (br), or a combination thereof. Coupling constants (J) are quoted to the nearest 0.5 Hz. Low resolution electrospray (ES) mass spectra were recorded on a Bruker Daltonics MicrOTOF mass spectrometer, run in positive mode. LC-MS analysis and chromatographic separation were conducted with a Brucker Daltonics MicrOTOF mass spectrometer or an Agilent Technologies 1200 series HPLC connected to an Agilent Technologies 6130 quadrupole LC/MS, where both instruments were connected to an Agilent diode array detector or on Bruker MicrOTOF II focus ESI Mass Spectrometer connected in parallel to Dionex Ultimate 3000 RSLC system with diode array detector. HPLC chromatographic separations were conducted using a Waters XBridge C18 column, 2.1 x 50mm, 3.5 µm particle size or Waters XSelect 2.1 x 30mm, 2.5 µm particle size. The compounds were eluted with a gradient of 5 to 95% acetonitrile/water +0.1% Ammonia or +0.1% formic acid.

Unless otherwise stated herein reactions have not been optimised. Solvents and reagents were purchased from commercial suppliers and used without further purification. Dry solvents were purchased in sure sealed bottles stored over molecular sieves.

The preparations and compounds have been named using the ChemDraw Ultra 12.0 naming application which is commercially available from the CambridgeSoft Corporation.

4-Oxochromene-2-carboxylic acid and 6-fluoro-4-oxo-chromene-2-carboxylic acid are commercially available from TCI-UK or Fluorochem.

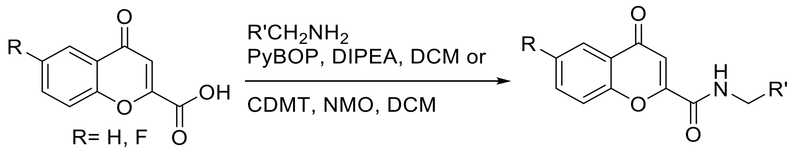

<u>*N*-(cyclohexylmethyl) 4-oxo-4*H*-chromene-2-carboxamide (2)</u>

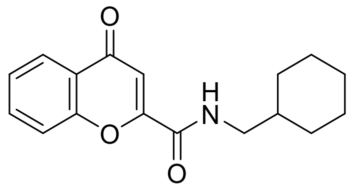

4-oxochromene-2-carboxylic acid (0.5 g, 2.6 mmol) was dissolved in DMF (6 ml) and diisopropylethyl amine (0.34 g, 2.6 mmol) was added. The reaction mixture was then cooled to 0 °C with an ice water bath and a solution of PyBOP in DCM (6 ml) was added. The mixture was stirred at 0°C for 30 min then cyclohexylmethanamine (298 mg, 2.6 mmol) was added and the reaction was stirred at room temperature for 4h. After solvent evaporation the crude material was purified by flash column chromatography eluting with 40 % EtOAc in heptane. The desired fractions were concentrated to dryness to give the desired product (256 mg, 35% yield). ^{1}H NMR (500 MHz, d^{6}-DMSO) 9.10 (1H, t, J=6.0 Hz), 8.06 (1H, dd, J=1.5, 7.9 Hz), 7.92 - 7.88 (1H, m), 7.76 (1H, d, J=8.2 Hz), 7.55 (1H, dd, J=7.1, 7.1 Hz), 6.83 (1H, s), 3.16 (2H, dd, J=6.6, 6.6 Hz), 1.71 (4H, t, J=13.5 Hz), 1.65 - 1.56 (2H, m), 1.25 - 1.14 (3H, m), 0.99 - 0.92 (2H, m) ppm. ^{13}C NMR (500 MHz, d^{6}-DMSO) δ 177.81, 159.51, 156.33, 155.61, 135.40, 126.46, 125.39, 124.13, 119.32, 110.88, 45.90, 37.82, 30.93, 26.46, 25.84 ppm. LC-MS (ESI) *m/z* 286 [M+H]+. HRMS (ES^{+}) calculated for $C_{17}H_{20}NO_3$ $[M+H]^+$= 286.1438, observed for $C_{17}H_{20}NO_3$ $[M+H]^+$= 286.1448.

<u>*N*-(cyclohexylmethyl)-6-fluoro-4-oxo-chromene-2-carboxamide (3).</u>

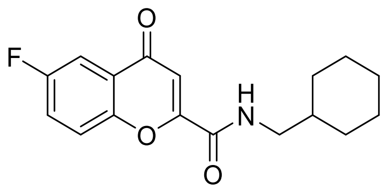

6-fluoro-4-oxo-chromene-2-carboxylic acid (0.13 mg, 0.62 mmol) was dissolved in DCM (5 ml) and diisopropylethyl amine (0.81 g, 0.62 mmol) was added. The reaction mixture was then cooled in an ice bath and a solution of PyBOP (0.32 g, 0.62 mmol) in DCM (2 ml) was added. The mixture was stirred at 0°C for 30 min and then the corresponding amine was added. The reaction mixture was stirred at room temperature for 4h. The crude was partitioned between DCM (10 ml) and brine (5 ml). The compound was precipitated from methanol and DMSO. Compound was filtered and dried to obtained *N*-(cyclohexylmethyl)-6-fluoro-4-oxo-chromene-2-carboxamide (20 mg, 10 % yield) as an off-white solid. ^{1}H NMR (500 MHz, $CDCl_3$) δ 7.87 (dd, J=3.1, 8.1 Hz, 1H), 7.56 (dd, J=4.1, 9.2 Hz, 1H), 7.47 (ddd, J=3.1, 7.4, 9.2 Hz, 1H), 7.17 (s, 1H), 6.88 (s, 1H), 3.36 (dd, J=6.6, 6.6 Hz, 2H), 1.84 - 1.75 (m, 3H), 1.74 - 1.62 (m, 2H), 1.56 (s, 1H), 1.33 - 1.18 (m, 3H), 1.08 - 0.99 (m, 2H) ppm. ^{13}C NMR (500 MHz, d^{6}-DMSO) δ 177.23, 160.67, 159.34, 156.55, 152.06, 125.39, 123.65, 122.18, 110.12, 109.96, 45.91,

37.83, 30.92, 26.45, 25.84 ppm. LC-MS (ESI) *m/z* 304 $(M+H)^+$. HRMS (ES^+) calculated for $C_{17}H_{18}FNO_3$ $[M+H]^+$= 304.1343, observed for $C_{17}H_{18}FNO_3$ $[M+H]^+$= 304.1349

6-Fluoro-*N*-[(1-hydroxycyclohexyl)methyl]-4-oxo-chromene-2-carboxamide (4).

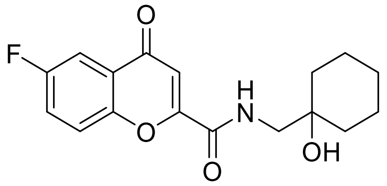

To a solution of 6-fluoro-4-oxo-chromene-2-carboxylic acid (0.5 g, 2.40 mmol) in DCM (10 ml) was added 2-chloro-4,6-dimethoxy-1,3,5-triazine (0.50 g, 2.88 mmol) followed by 4-methylmorpholine (0.97 g, 9.61 mmol) and the mixture stirred for 30 min. 1-(aminomethyl)cyclohexanol hydrochloride (0.60 g, 3.60 mmol) was then added in one portion and the mixture stirred at room temperature overnight. The mixture was then washed with $NaHCO_3$ saturated aqueous solution (5 ml). Organic phase was evaporated to dryness and the residue was purified by SCX column (2 g) and product was eluted with MeOH. Methanol filtrate concentrated under reduced pressure. Product was purified by preparative HPLC eluting with a solution 0.1% HCO_2H in water and acetonitrile. Fractions containing product were pooled together to obtain 6-fluoro-N-[(1-hydroxycyclohexyl)methyl]-4-oxo-chromene-2-carboxamide (215 mg, 27% yield) as a white solid. ^1H NMR (500 MHz, $CDCl_3$) δ 7.83 (dd, J=3.1, 7.9 Hz, 1H), 7.60 - 7.56 (m, 1H), 7.46 (ddd, J=3.1, 7.5, 9.2 Hz, 1H), 7.37 (t, J=5.5 Hz, 1H), 7.16 (s, 1H), 3.54 (d, J=6.1 Hz, 2H), 2.22 - 2.15 (m, 1H), 1.66 - 1.53 (m, 9H), 1.41 - 1.34 (m, 1H) ppm. ^{13}C NMR (500 MHz, d^6-DMSO) δ 177.21, 177.19, 160.68, 159.70, 158.73, 156.56, 152.05, 152.04, 125.37, 125.31, 123.64, 123.44, 122.27, 122.20, 110.31, 110.14, 109.95, 70.81, 49.95, 35.45, 25.89, 21.93 ppm. LC-MS (ESI) *m/z* 320 $(M+H)^+$. HRMS (ES^+) calculated for $C_{17}H_{19}FNO_4$ $[M+H]^+$= 320.1293, observed for $C_{17}H_{19}FNO_4$ $[M+H]^+$= 320.1290.

***N*-[(4,4-difluoro-1-hydroxy-cyclohexyl)methyl]-6-fluoro-4-oxo-chromene-2-carboxamide (5).**

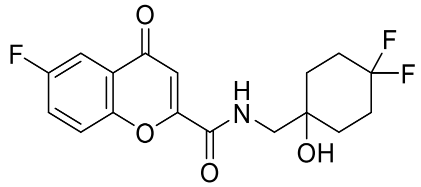

To a solution of 6-fluoro-4-oxo-chromene-2-carboxylic acid (0.40 g, 1.92 mmol) in DCM (20 ml) was added 2-chloro-4,6-dimethoxy-1,3,5-triazine (0.40 g, 2.3 mmol) followed by 4-methylmorpholine (0.78 g,7.69 mmol) and the mixture stirred for 30 min. 1-(Aminomethyl)-4,4-difluoro-cyclohexanol hydrochloride (0.53 g, 2.65 mmol) was then added in one portion and the mixture stirred at room temperature overnight. The mixture was then washed with $NaHCO_3$ saturated aqueous solution (5 ml). Organic phase was evaporated to dryness and the residue was purified by SCX column (10 g) and product was eluted with MeOH. Methanol filtrate concentrated under reduced pressure. Product was purified by preparative HPLC eluting with a solution of 0.1% HCO_2H in water and acetonitrile. Fractions containing product were pooled together to obtain *N*-[(4,4-difluoro-1-hydroxy-cyclohexyl)methyl]-6-fluoro-4-oxo-chromene-2-carboxamide (0.39 mg, 56 % yield) as a white solid. ^1H NMR (500 MHz, DMSO) δ 8.99 (t, J=6.2 Hz, 1H), 7.89 - 7.73 (m, 3H), 6.88 (s, 1H), 4.80 (s, 1H), 3.37 (d, J=6.4 Hz, 2H), 2.10 - 1.86 (m, 4H), 1.68 - 1.53 (m, 4H) ppm. ^{13}C NMR (500 MHz, DMSO) δ 159.90,

156.41, 125.32, 123.72, 123.52, 122.24, 110.38, 110.17, 109.99, 69.69, 49.45, 31.66, 31.59, 29.50 ppm. LC-MS (ESI) *m/z* 356 $(M+H)^+$. HRMS (ES^+) calculated for $C_{17}H_{17}F_3NO_4$ $[M+H]^+$= 356.1104, observed for $C_{17}H_{17}F_3NO_4$ $[M+H]^+$= 356.1110.

2. Biochemistry

2.1 Protein expression and purification method for recombinant KRS (UW)

Recombinant KRS enzymes were cloned and expressed as previously described (1). Briefly, PCR amplicons were generated from the following template sources and primers:

P. falciparum 3D7 cDNA
(GGGTCCTGGTTCGATGGAAGTGGATCCAAGATTATATTT
and CTTGTTCGTGCTGTTTATTAATTTGCTGGTCGCATAGTGGGAA),

C. parvum Iowa II cDNA (GGGTCCTGGTTCGATGCACTATACTGATAATAGGTACAA
and CTTGTTCGTGCTGTTTATTAATTTCCGCTATGTTGGTTGCTATG),

human pET28a-KRS construct (Medicinal Bioconvergence Research Center, Seoul, South Korea)
(GGGTCCTGGTTCGATGGCGGCCGTGCAGGCGG
and CTTGTTCGTGCTGTTTATTAGACAGAAGTGCCAACTGTTGTGC).

The *P. falciparum* KRS had 76 residues removed at the *N*-terminus and replaced with maltose binding protein (MBP) to improve solubility. The *C. parvum* KRS had 45 residues removed at the *N*-terminus. The human KRS was the full length, untruncated sequence. All amplicons were cloned into the pAVA0421 expression vector using the ligation independent cloning (LIC) method. Recombinant plasmids were transformed into Rosetta BL21(DE3) competent cells and plated on LB agar plates with ampicillin, carbenicillin, and chloramphenicol selection. Clones were expressed using previously reported autoinduction methods and scaled to 2-litre cultures in a LEX bioreactor for large scale protein purification. Cell pellets were harvested and lysed with CHAPS detergent buffer and centrifuged to remove cell debris. The expressed products included a 6x-HIS tag appended to the *N*-termini for IMAC purification. Subsequent size exclusion chromatography was employed to further purify the recombinant proteins (2).

2.2 Protein expression and purification method for recombinant *Pf*KRS1 (Dundee)

The gene coding for *P. falciparum* KRS1 80-583, codon optimized for expression *in E.coli*, obtained from Genscript, was inserted into a His MBP TEV pET15b vector using Nde1 and Xho1 restriction sites. The resulting expression construct was introduced into BL21 (DE3) competent cells for protein production. A 120 ml overnight culture was set up and grown at 37°C 200 rpm for 16 h and was used the next day to inoculate 12 litres of Autoinduction+AMP media. Cells were grown at 37°C for 4 h then 20°C for 20 h before harvesting by centrifugation at 3,500 g for 30 min and storage at -20°C. The resulting cell pellet was 65 g. Lysis buffer (200 ml, 25 mM TRIS/500 mM NaCl/20 mM Imidazole pH 8.5/protease inhibitor tablets/DNAase) was added and the pellets defrosted at 25°C in a water bath

for approximately 20 min. The slurry was then passed through a Cell Disrupter (Constant Systems) set at 30 KPSI to lyse the cells. The sample was then centrifuged at 40,000g for 20 min. The supernatant was then filtered using syringe filters to 0.2 µm. The supernatant was loaded onto a 5 ml HiTrap Ni HP column that had been equilibrated with Buffer A (25 mM TRIS/500 mM NaCl/20 mM Imidazole pH 8.5) at 5 ml/min using an AKTA Pure system. Once loaded the column was washed with 10 column volumes of buffer A followed by 5% Buffer B (25 mM TRIS/500 mM NaCl/500 mM Imidazole pH 8.5) to wash off His-rich contaminating proteins. A linear gradient of 5-50% B was used to elute the protein. Approximately 130 mg of protein was present in the fractions containing the *Pf*KRS1 protein. The sample was then passed through a 0.2 µm filter, before loading onto a XK50/60 Superdex 200 column using an AKTA Pure system at 4°C at 3 ml/min. The eluted protein was buffer exchanged into KRS Buffer (50 mM TRIS/200 mM NaCl/ 10 µM 2-mercaptoethanol pH 8.0) and concentrated to 7.75 mg/ml.

2.3 Protein expression and purification method for recombinant *Cp*KRS (Dundee)

The *C. parvum* KRS had 45 residues removed at the N-terminus. All amplicons were cloned into the pAVA0421 expression vector using the ligation independent cloning (LIC) method. Recombinant plasmids were transformed into Rosetta BL21(DE3) competent cells and plated on LB agar plates with ampicillin, carbenicillin, and chloramphenicol selection. Clones were expressed using previously reported autoinduction methods and scaled to 2-litre cultures in shake flasks. Cell pellets were harvested and lysed with 100 mM HEPES/150 mM NaCl/5% Glycerol/20 mM imidazole/0.5 mM TCEP pH 7.5/DNase/Complete inhibitor tablets) using a Cell Disrupter (Constant Systems) at 30 KPSI and centrifuged at 40,000g for 20 min remove cell debris. The supernatant was loaded onto a 5 ml HiTrap Ni HP column that had been equilibrated with Buffer A (100 mM HEPES/150 mM NaCl/5% Glycerol/20mM imidazole/0.5 mM TCEP pH 7.5) at 5 ml/min using an AKTA Pure system. Once loaded the column was washed with 10 column volumes of buffer A. A 5% step of Buffer B (100 mM HEPES/150mM NaCl/5% Glycerol/500 mM imidazole/0.5 mM TCEP pH 7.5) was then used to wash off His-rich contaminating proteins. A linear gradient of 5-50% B was used to elute the protein. Approximately 287 mg of protein was present in the fractions containing the *Cp*KRS protein. The sample was then concentrated to approximately 30 ml, passed through a 0.2 µm filter, before loading onto a XK26/60 Superdex 200 column using an AKTA Pure system at 4°C at 2 ml/min 10 ml at a time. The eluted protein was pooled then frozen at -80°C. The concentration of the protein was 1.3 mg/ml. Sometimes the protein was cleaved with PreScission protease overnight, a second Ni column performed in flow through mode to removed uncleaved protein. GST beads were then added to remove the PreScission Protease. The protein was then gel filtered as descripted above.

2.4 Protein expression and purification method for recombinant *Hs*KRS (Dundee)

The gene coding for human KRS, codon optimized for expression *in E. coli,* was obtained from Genscript and cloned into a His PP pET15b vector using Nde1 and Xho1 restriction sites. The resulting vector was used to transform BL21 (DE3) competent cells for protein expression. A 120 ml overnight culture was set up and grown at 37°C 200 rpm for 16 h and used the next day to inoculate 6 litres of Autoinduction+AMP medium. This culture was grown at 37°C for 4 h then 20°C for 20 h. The cells were harvested by centrifugation at 3,500 g for 30 min then stored at -20°C. The resulting cell pellet was 50 g. Lysis buffer (150 ml, 25 mM HEPES/500 mM NaCl/20 mM imidazole/ 2mM DTT/10%

glycerol pH 7.5/protease inhibitor tablets/DNAase) was added and the pellets for defrosted at room temperature for approximately 10 min. The slurry was then passed through a Cell Disrupter (Constant Systems) set at 30 KPSI to lyse the cells. The sample was then centrifuged at 40,000g for 20 min. The supernatant was then filtered using syringe filters to 0.45 µm. The supernatant was loaded onto a 5 ml HiTrap Ni HP column that had been equilibrated with Buffer A (25 mM HEPES/500 mM NaCl/20 mM Imidazole/2 mM DTT/10% Glycerol pH 7.5) at 5 ml/min using an AKTA Pure system. Once loaded the column was washed with 10 column volumes buffer A. A 5% step of Buffer B (25 mM HEPES/500 mM NaCl/500 mM Imidazole/2mM DTT/10% Glycerol pH 7.5) was then used to wash off His rich contaminating proteins. A gradient of 5-50% B was used to elute the protein. Approximately 55 mg of protein was present in the fractions containing the *Hs*KRS protein. The sample was then passed through a 0.2 µm filter, before loading onto a XK26/60 Superdex 200 column equilibrated with Buffer C (25 mM HEPES/150 mM NaCl /2mM DTT/10% glycerol pH 7.0) using an AKTA Pure system at 4°C at 2 ml/min. The sample was then dialyzed into 25 mM HEPES/500 mM NaCl/2 mM DTT/10% glycerol/0.25% azide pH 7.0. The protein was concentrated to 1 mg/ml and frozen at -80°C. Approximately 23 mg of protein was produced.

2.5 *Pf*KRS1, *Cp*KRS and *Hs*KRS Pyrophosphate Generation EnzChek^{TM} Kinetic Measurements

Steady-state kinetic measurements were conducted at room temperature using a PheraStar plate reader (BMG). End-points for the aminoacylation reaction of *Pf*KRS1, which produces inorganic pyrophosphate that is converted to two molecules of inorganic phosphate using a pyrophosphatase enzyme, were measured using the EnzChek™ Phosphate Assay Kit, which gives an absorbance read-out at 360 nm. Typical end-point assays were carried out in clear, flat-bottom, polystyrene, 384-well plates (Greiner) in an 80 µl reaction volume containing 100 mM Hepes, pH7.4; 100 mM NaCl; 20 mM $MgCl_2$; 1 mM DTT; 0.05% IGEPAL®; 0.5 U/ml pyrophosphatase; 0.1 mM MESG; 0.5 U/ml PNP; 0.5 mM ATP; 5 mM L-lysine and 100, 300 or 400 nM recombinant *Pf*KRS1, *Cp*KRS or *Hs*KRS respectively. Assays were performed by adding 40 µl of a 2-times concentrated reaction mixture containing all components with the exception of the substrates to all wells, and the reactions started by adding 40 µl of a 2-times concentrated substrate mixture. The reaction was carried out for 6 h (*Pf*KRS1 and *Cp*KRS) or 12 h (*Hs*KRS) at room temperature and then the absorbance of each well was read at 360 nm. The substrates K_m values were determined by varying the concentration of one of the substrates at saturating concentration of the other. Due to limiting assay conditions, *i.e.* low enzyme activity and high affinity substrates, for *Hs*KRS, only apparent K_m values could be obtained.

Steady-state data were fitted using the nonlinear, least-squares, curve-fitting programs of Sigma-Plot for Windows, version 12.5. Individual saturation curves were fit to eq. 1

$$v = \frac{VK}{K+S} \qquad (1)$$

where V is the maximal velocity (here expressed as µM/min of phosphate production), S is the substrate concentration and K is the Michaelis constant for the substrate (K_m). Individual saturation curves displaying cooperativity were fit to eq. 2

$$v = \frac{VK^n}{K^n + S^n} \qquad (2)$$

where *n* is the Hill slope.

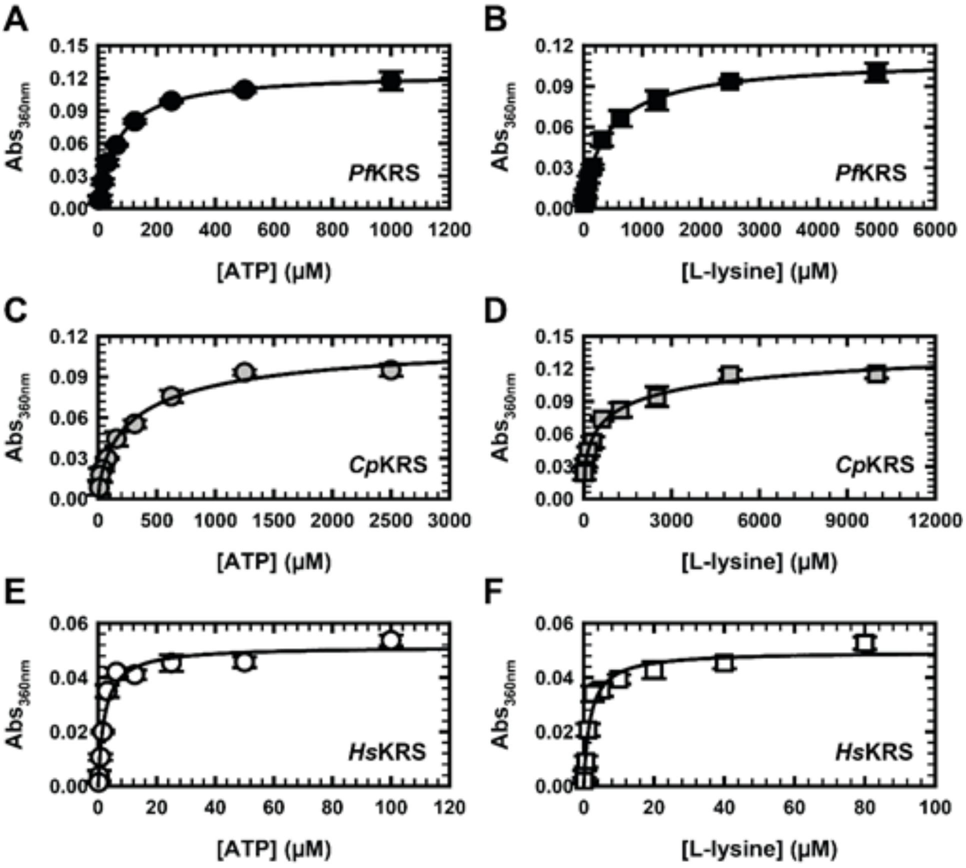

Figure S1. Steady-state kinetics of *Pf*, *Cp* and *Hs*KRS (EnzChek).
*Pf*KRS1 saturation curves for (A) ATP and (B) L-lysine, *Cp*KRS saturation curves for (C) ATP and (D) L-lysine, and *Hs*KRS saturation curves for (E) ATP and (F) L-lysine. Points are data and error bars indicate the standard deviation, n = 3. Lines are the best fit to eq. 1 (A, E and F) and eq. 2 (B, C and D).

Table S1. Steady-state kinetic parameters for *Pf*, *Cp* and *Hs*KRS (EnzChek).

Enzyme	Parameter	Best fit	Equation
***Pf*KRS1**	$K_{m,ATP}$ (μM)	68 ± 3	1
	$K_{m,L\text{-}Lys}$ (μM)	413 ± 37	2
	$Hill_{,L\text{-}Lys}$	0.89 ± 0.04	2
***Cp*KRS**	$K_{m,ATP}$ (μM)	346 ± 128	2
	$Hill_{,ATP}$	0.71 ± 0.09	2
	$K_{m,L\text{-}Lys}$ (μM)	1045 ± 640	2
	$Hill_{,L\text{-}Lys}$	0.49 ± 0.06	2
***Hs*KRS**	$K_m^{app}{}_{,ATP}$ (μM)	2.22 ± 0.44	1
	$K_m^{app}{}_{,L\text{-}Lys}$ (μM)	1.92 ± 0.37	1

2.6 *Pf*KRS1 Hit Discovery Using Luciferase ATP Consumption (Kinase Glo®) (UW)

An initial *Pf*KRS1 high-throughput screen of the 'TCAMS *set',* a library of ~13,000 compounds which show phenotypic activity against *P. falciparum* (provided by GlaxoSmithKline) (3), was performed using the Kinase-Glo® assay platform (Promega, Madison, WI). Assays were performed in 384-well format in buffer (as described in Table S3), 60 µM lysine, and 75 nM *Pf*KRS1. Compounds were initially screened at single point concentrations of 7.5 µM and hits were repeated three times. Plates were incubated for 180 mins at 37°C after addition of compounds. Reactions were terminated by the addition of Kinase-Glo® reagent and read on an EnVision plate reader (PerkinElmer, Waltham, MA). Compound **2** was identified as a top hit with 71-91% inhibition.

2.7 *Pf*KRS1, *Cp*KRS and *Hs*KRS SAR Assay Using Luciferase ATP Consumption (Kinase Glo®)

Inhibition of KRS activity by cladosporin and other compounds was assayed in the buffered medium. Assays were run in 96-well white flat-bottom polystyrene NBS™ Microplates (Corning Inc., Corning, NY) in a final reaction volume of 50 µl. Each assay well was composed of buffer (as described in Table S3), 10 µM lysine and either 8.1 nM *Pf*KRS1, 8.1 nM *Cp*KRS or 6.6 nM *Hs*KRS. Enzymes were pre-incubated with compound before the remaining assay reagents were added. The reaction was initiated by the addition of 2 µM ATP. The plate was incubated with shaking at 90 rpm and 30°C for 120 min. Reactions were terminated by the addition of 50 µl Kinase-Glo® reagent (Promega, Madison, WI). Maximum and minimum signal controls were included in each reaction plate. The reaction was read on a MicroBeta2® plate reader (PerkinElmer, Waltham, MA). Assays were performed independently three times. Analysis and calculation of inhibitors IC_{50} values were performed using Graphpad® Prism software (GraphPad Software, San Diego, CA).

2.8 *Pf*KRS1 Mode of Inhibition Studies by Compound 5 Using Pyrophosphate Generation (EnzChek™)

Using the EnzChek assay platform the IC_{50} value for compound **5** was determined at saturating concentrations of both substrates, 0.5 mM ATP and 5 mM L-lysine, in a series of 10 half-log dilutions of compound **5**. To determine the steady-state inhibition parameters and patterns associated with compound **5**, *Pf*KRS1 activity was studied in the presence of variable concentrations of one substrate, ATP or L-lysine, fixed saturating concentration of the co-substrate (L-lysine or ATP) and several fixed concentrations of compound **5**. Assay ready plates containing different concentrations of compound **5** were prepared using an Echo® 550 acoustic dispenser (Labcyte Inc.). The assays were carried out as described above in section 2.5.

Inhibition data obtained under saturating concentrations of substrates, and variable concentration of compound **5** were fit to eq. 3

$$y = y_{min} + \frac{y_{max} - y_{min}}{1 + \left(\frac{IC_{50}}{I}\right)^n} \qquad (3)$$

Where y_{min} corresponds to no inhibition and y_{max} to maximum inhibition, IC_{50} is the concentration of inhibitor necessary to give 50% inhibition and I is the inhibitor concentration. Inhibition data showing linear competitive patterns in double-reciprocal plots were fit to eq. 4

$$v = \frac{VS}{K\left(1+\frac{I}{K_i}\right)+S} \qquad (4)$$

where K_i is the dissociation constant for the enzyme-inhibitor complex. Inhibition data showing linear, uncompetitive patterns in double-reciprocal plots were fit to eq. 5

$$v = \frac{VS}{K+S\left(1+\frac{I}{K_i}\right)} \qquad (5)$$

Table S2. Steady-state kinetic parameters for *Pf*KRS1.

Parameter	Best fit	Equation
**IC_{50} (μM)**	0.210 ± 0.012	3
**$K_{i,ATP}$ (μM)**	0.032 ± 0.004	4
**$K_{i,L\text{-}lys}$ (μM)**	0.212 ± 0.014	5

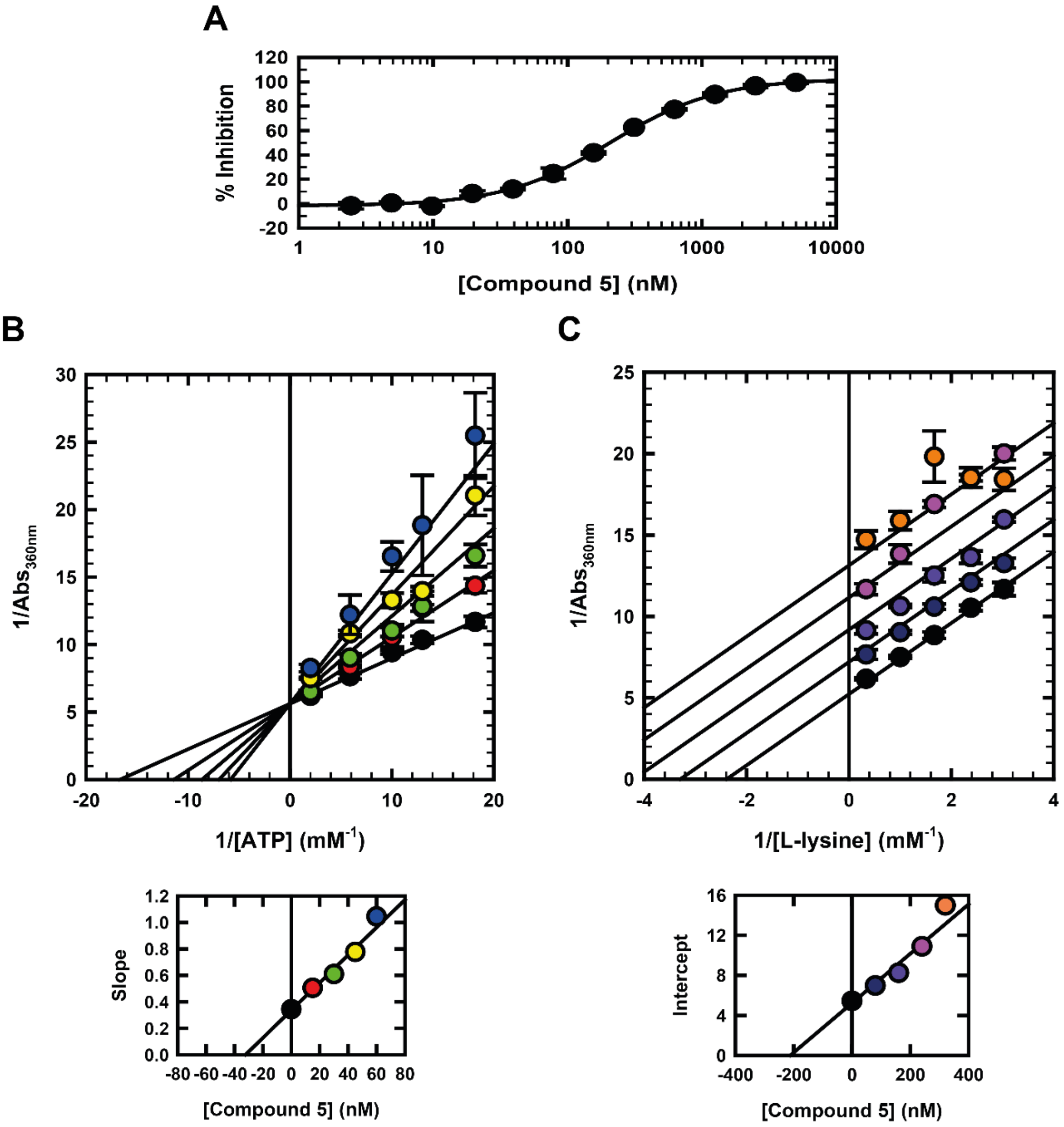

Figure S2. Steady-state kinetics of *Pf*KRS1 inhibition by compound 5.

(A) Saturation curve for (A) Compound **5**. Points are data and error bars indicate the standard deviation, n = 3. The line is the best fit to eq. 3. (B) Double-reciprocal plot illustrating the linear competitive inhibition pattern obtained when varying the concentration of compound **5** at fixed variable concentrations of ATP and saturating concentrations of L-lysine. Points are data obtained with 0 (black circles), 15 (red circles), 30 (green circles), 45 (yellow circles) and 60 nM (blue circles) of compound **5**. The error bars indicate the standard deviation, n = 3. Lines are the best fit of the entire data set to eq. 4. The bottom figure is a replot of the slopes of the linear regression of the data at each concentration of compound **5**, showing the linear dependence on inhibitor concentration. Points are data, and the line is the linear regression of the data. (C) Double-reciprocal plot illustrating the linear uncompetitive inhibition pattern obtained when varying the concentration of compound **5** at fixed variable concentrations of L-lysine and saturating concentrations of ATP. Points are data obtained with 0 (black circles), 80 (dark blue circles), 160 (violet circles), 240 (pink circles) and 320 nM (orange circles) of compound **5**. The error bars indicate the standard deviation, n = 3. Lines are the best fit of the entire data set to eq. 5. The bottom figure is a replot of the intercepts of the linear regression of the data at each concentration of compound **5**, showing the linear dependence on inhibitor concentration. Points are data, and the line is the linear regression of the data.

2.9 Recombinant KRS thermal shift assays (UW)

Recombinant *Pf*KRS1, *Cp*KRS, and *Hs*KRS were diluted to 4 μM in assay buffer containing 20 mM Tris-HCl pH 8, 200 mM NaCl, 5 mM DTT, 5 mM $MgCl_2$ and ligands (1 mM lysine; 40 μM ATP; and 40 μM inhibitors) in various combinations. Aliquots (10 μl) were added to a 96-well PCR plate and incubated at ambient temperature for 15 min. SYPRO® Orange dye (Invitrogen) was diluted in assay buffer to a 5x concentration and 10 μl was added to the PCR plate to bring the final assay volume to 20 μl. The plate was sealed and pulse centrifuged to consolidate the assay mixture and remove air bubbles. Using a StepOne Plus RT-PCR thermal cycler (Applied Biosystems) to monitor fluorescence at an excitation/emission wavelength of 300/470-570 nm, the samples were heated from 25°C to 99°C at a ramp rate of .016°C/sec. Graphpad® Prism software (GraphPad Software, San Diego, CA) was used to determine melting temperatures (T_m) using Boltzmann sigmoidal analysis.

Table S3. Thermal shift data for compounds *with PfKRS1, CpKRS and HsKRS.*

Compound Effects on LysRS Melting Temperature (ΔT_m)

**Compound ID**	Initial T_ms (°C)			**1**			**2**			**5**			**3**			**4**		
	***Pf*KRS**	***CpK* RS**	***HsK* RS**	***PfK* RS**	***CpK* RS**	***HsK* RS**	***PfK* RS**	***Cp* KRS**	***HsK* RS**	***PfK* RS**	***Cp* KRS**	***HsK* RS**	***PfK* RS**	***Cp* KRS**	***HsK* RS**	***PfK* RS**	***Cp* KRS**	***HsK* RS**
	45.7	***42.5***	***60.2***	3.4	-0.1	-0.1	1.2	0.0	-0.3	1.3	-0.5	0.3	0.5	-0.2	-0.1	0.9	-0.2	-0.5
**ATP**	***45.9***	***42.1***	***59.9***	3.5	0.1	0.3	0.9	0.7	0.3	0.9	-0.2	0.4	0.2	0.2	0.4	0.5	0.0	-0.1
**Lysine**	***48.4***	***43.6***	***59.5***	9.3	19.5	-9.2	7.4	19.3	-10.0	7.5	14.7	-7.6	5.3	1.2	-8.4	7.5	18.4	-7.4
**ATP + Lysine**	***50.7***	***46.1***	***60.2***	7.2	14.0	-8.2	5.1	11.7	-8.6	5.3	13.0	-7.4	2.4	0.8	-10.8	5.1	11.8	-7.2

3. Cell Biology

3.1 Assay methodology for *Plasmodium falciparum* (3D7) (Dundee).

Cultures of the widely-used malaria reference strain of chloroquinine-sensitive *Plasmodium falciparum* strain 3D7 were maintained in a 5% suspension of human red blood cells cultured in RPMI 1640 medium supplemented with 0.5% Albumax II (available from Gibco Life Technologies, San Diego, CA, cat.no. 11021-037), 12 mM sodium bicarbonate, 0.2 mM hypoxanthine, (pH 7.3), and 20 mg/litre gentamicin at 37°C, in a humified atmosphere of 1% O_2, 3% CO_2 with a gas balance of nitrogen.

Growth inhibition of the *Plasmodium falciparum* cultures was quantified in a 10-point dose response curve with a 1 in 3 dilution series from a top assay concentration of 50μM. This 384 well plate based fluorescence assay utilises the binding of SYBRgreen I (Thermo Fisher Scientific/Invitrogen cat.no. S7585) to double stranded DNA, which greatly increases the fluorescent signal at 528 nm after excitation at 485 nm. Mefloquine was used as a drug control to monitor the quality of the assay (*Z′* = 0.6 to 0.8, where *Z′* is a measure of the discrimination between the positive and negative controls on a screen plate). Dose-response curves were determined from a minimum of 3 independent experiments. Compound bioactivity was expressed as EC_{50}, the effective concentration of compound causing 50% parasite death. EC_{50} values were determined from a minimum of 3 independent experiments.

3.2 Cytotoxicity Studies (Dundee)

In vitro cytotoxicity studies can be carried out using Hep G2 (Human Caucasian hepatocyte carcinoma, ECACC cat.no. 85011430) used as indicators for general mammalian cell toxicity. Hep G2 *in vitro* cytotoxicity can be assessed using the assay procedure as described (4).

3.3 *In vitro* Cell Assay Data Analysis (Dundee)

All data was processed using IDBS ActivityBase® raw data was converted into per cent inhibition through linear regression by setting the high inhibition control as 100% and the no inhibition control as 0%. Quality control criteria for passing plates were as follows: Z'> 0.5, S:B> 3, $\%CV_{(\text{no inhibition control})} < 15$. The formula used to calculate Z' is $1-\frac{3\times(StDev\_high+StDev\_low)}{ABS(Mean\_high-Mean\_low)}$.

All EC_{50} Curve fitting was undertaken using XLFit version 4.2 using Model 205 with the following 4 parametric equation: $A+\frac{(B\text{-}A)}{(1+(C/x)^{D})}$,where A=% inhibition at bottom, B=% inhibition at top, C= EC_{50}, D= slope, *x*= inhibitor concentration and y= % inhibition. If curve did not reach 100% of inhibition, B was fixed to 100 only when at least 50% of inhibition was reached.

3.4 Assay methodology for drug resistant *Plasmodium falciparum* (K1 and TM90C2B) (SwissTPH).

Plasmodium falciparum drug-sensitive NF54 (airport strain from The Netherlands, provided by F. Hoffmann-La Roche Ltd) and resistant strains K1 and TM90C2B (from the Malaria Research and Reference Reagent Resource Center, accurate descriptions of the genetic backgrounds of these strains can be obtained at http://www.beiresources.org) were cultivated in a variation of the medium previously described (5, 6), consisting of RPMI 1640 supplemented with 0.5% ALBUMAX® II, 25 mM Hepes, 25 mM $NaHCO_3$ (pH 7.3), 0.36 mM hypoxanthine, and 100 µg/ml neomycin. Human erythrocytes served as host cells. Cultures were maintained in an atmosphere of 3% O_2, 4% CO_2, and 93% N_2 in humidified modular chambers at 37 °C. Compounds were dissolved in DMSO (10 mM), diluted in hypoxanthine-free culture medium and titrated in duplicates over a 64-fold range in 96 well plates. Infected erythrocytes (1.25% final hematocrit and 0.3% final parasitemia) were added into the wells. After 48 h incubation, 0.25 µCi of $[^3H]$-hypoxanthine was added per well and plates were incubated for an additional 24 h. Parasites were harvested onto glass-fiber filters and radioactivity was counted using a Betaplate liquid scintillation counter (Wallac, Zurich). The results were recorded and expressed as a percentage of the untreated controls. Fifty percent inhibitory concentrations (EC_{50}) were estimated by linear interpolation (7).

3.5 *In vitro* parasite reduction ratio (PRR) assay with *Plasmodium falciparum (GSK)*

The *in vitro* Parasite Rate Reduction assay (PRR) was conducted as previously described (8). Briefly, *Plasmodium falciparum* parasites were exposed to compound **5** for 120 h at a concentration corresponding to 10 x EC_{50}. Drug was renewed daily over the entire treatment period. Samples of parasites were taken from the treated culture at intervals (24, 48, 72, 96 and 120 h time points), drug was washed out and drug-free parasites were cultured in 96-well plates by adding fresh erythrocytes

and new culture media. The number of viable parasites was determined by the serial dilution technique. Four independent serial dilutions were done with each sample to correct for experimental variation.

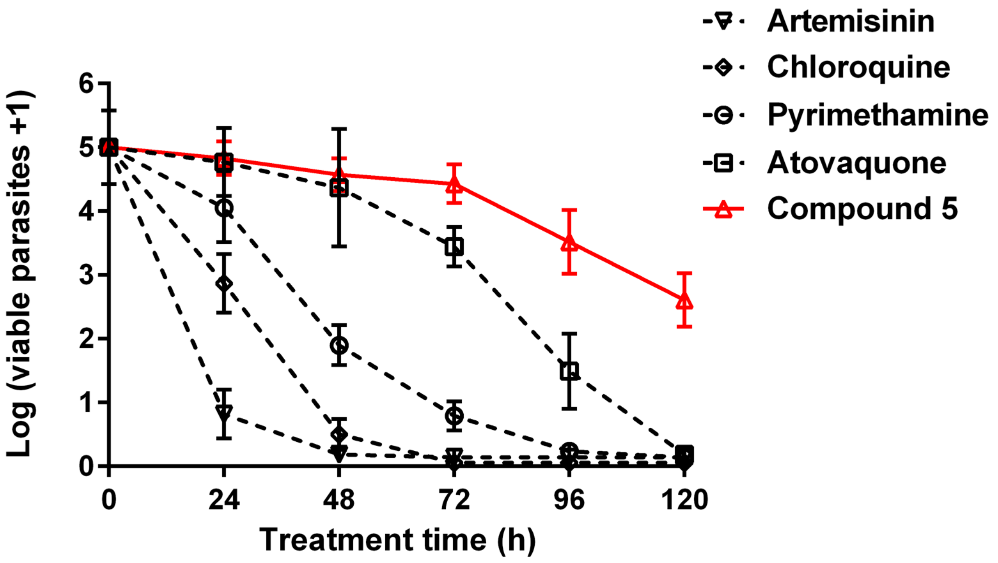

Figure S3. The rate of kill of *P. falciparum* by compound **5**. Data for standard drugs are provided for comparison as previously reported (8).

3.6 Inhibition of Nanoluciferase expressing *C. parvum* growth in HCT-8 cells. (UW)

Inhibition of *C. parvum* growth and EC_{50} determinations were performed with a Nanoluciferase-expressing *C. parvum* parasite in HCT-8 cells. Oocysts were propagated in calves (*Cryptosporidium* Production Laboratory, School of Animal and Comparative Biomedical Sciences, University of Arizona) and isolated from stools by sucrose suspension and cesium chloride gradient. HCT-8 cells were seeded to a 384-well plate and allowed to grow for 72 h to reach 90-100% confluence at 37°C in 5% CO_2. Oocysts were activated by 10-15 min incubation in 10% bleach (0.6% sodium hypochlorite) at room temperature and then washed with DPBS. 2000 oocysts per well were applied to 384-well plates with RPMI-1640 medium supplemented with 10% horse serum and 1% penicillin/streptomycin at the same time as compound addition. The compound and the oocysts were left in place for the full 72 h growth time. The amount of luciferase-induced light emission was determined by lysing the cell monolayer, adding Nano-Glo® luciferase reagent (Promega, Madison, WI, USA), and reading on an EnVision Multilabel Plate Reader (Perkin Elmer, Waltham, MA, USA). Controls included infected wells with no addition of inhibitors and the background was taken as wells where no *C. parvum* was added. The percentage of growth versus infected cells with no inhibitor was calculated for each concentration of BKI and the half maximal effective concentration (EC_{50}) values were determined by a sigmoidal dose response with variable slope using GraphPad Prism version 6.07 (GraphPad Software, La Jolla, California, USA).

3.7 Assay method for measurement of *in vitro* inhibition of *Cryptosporidium*. (Vermont)

The *in vitro* measurement of EC_{50}, the effective concentration of compound causing 50% parasite death, versus *Cryptosporidium* were run in accordance with the method of Bessoff *et.al.* (9) Human

ileocecal adenocarcinoma (HCT-8) cells (ATCC) were cultured in RPMI 1640 medium (Invitrogen) supplemented with 10% fetal bovine serum (Sigma-Aldrich), 120 U/ml penicillin, and 120 µg/ml streptomycin (ATCC), and used between passages 9 and 39 for all experiments. *Cryptosporidium parvum* Iowa isolate oocysts were purchased from Bunch Grass Farm (Deary, ID). Oocysts were triggered to excyst by treating with 10 mM hydrochloric acid (10 min, 37°C) and then 2 mM sodium taurocholate (Sigma-Aldrich) in PBS (10 min, 16°C). They were then added to >95% confluent HCT-8 cell monolayers in 384-well plates (~5,500 oocysts/well). Experimental compounds were added 3 h after infection, and assay plates were incubated for 48 h post-infection at 37°C under 5% CO_2. Assay plates were then washed three times with PBS containing 111 mM D-galactose, fixed with 4% paraformaldehyde in PBS, treated for 10 min at 37°C with 0.25% Triton X-100, and stained with 1.33 µg/ml of fluorescein-labeled *Vicia villosa* lectin (Vector Laboratories) and Hoechst 33258 (AnaSpec) at a final concentration of 0.09 mM. Epifluorescent images were acquired using a Nikon Eclipse TE2000 microscope with an automated stage programmed to focus on the center of each well and acquire a 3-by-3 composite image using an Exi Blue camera (QImaging, Canada) and a 20× objective (numerical aperture, 0.45). Nucleus and parasite images were exported separately as .tif files, and parasites and host cells were enumerated using macros developed for NIH ImageJ. Curves were plotted and the half-maximal effective (EC_{50}) and 90% effective concentration (EC_{90}) were calculated using GraphPad Prism software, version 6.01.

3.8 *In vitro* Cryptosporidium time-kill curve assay (Vermont)

Cryptosporidium parvum elimination during culture in HCT-8 cells was measured as recently described (10). Excysted *C. parvum* oocysts were added to HCT-8 cells in 384-well plates. Compounds were added 24 h after infection at 1, 3, 6, 9, and 12× the EC_{90}, followed by incubation for an additional 0, 6, 12, 24, 48, or 72 h before preparation, staining, and measurement of parasite and host cell numbers by epifluorescence microscopy. GraphPad Prism software was used to fit parasite decay curves to the number of parasites expressed as the percentage of parasites in vehicle-treated (DMSO) wells.

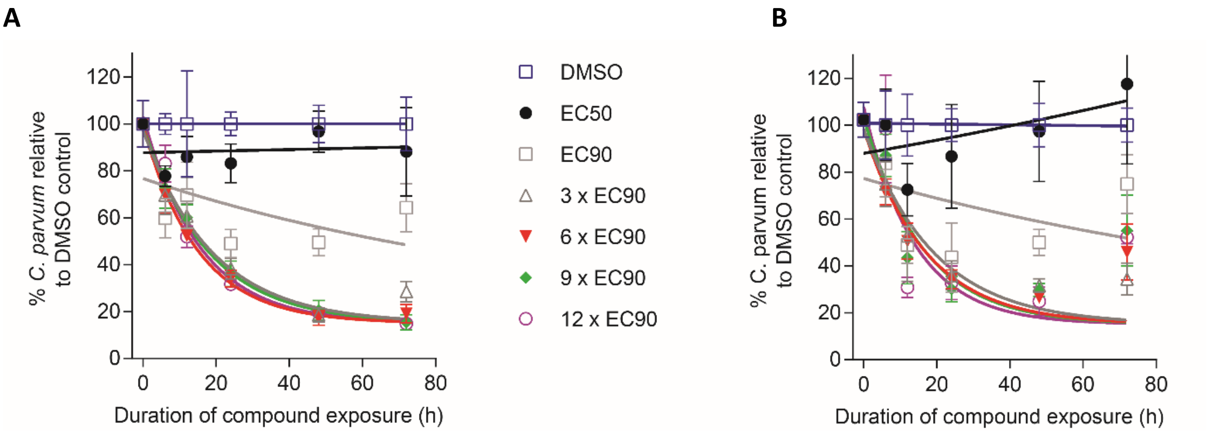

Figure S4. The rate of kill of *C. parvum* by cladosporin (A) and compound **5** (B).

3.9 *Plasmodium vivax* liver schizonts/ hypnozoites (University of Georgia)

This is based on methods previously reported (11)

3.10 *Plasmodium berghei* liver schizonts (UCSD)

This is based on methods previously reported (12). In brief, HepG2-A16-CD81EGFP cells were cultured at 37 °C in 5% CO2 in DMEM media. For both *P. berghei*-Luc and HepG2 cytotoxicity assays, 3x10**3 of the HepG2-A16-CD81EGFP20 cells in 5 µl of assay medium (DMEM without Phenol Red (Life Technologies, CA), 5% FBS, and 5x Pen Strep Glutamine (Life Technologies, CA)) at concentration 6x10**5 cells/ml were seeded in white solid bottom 1536-well plates (custom GNF mold ref# 789173-F, Greiner Bio-One), 20-26 h prior to sporozoites infection. For IC50 determinations, 18 h prior to infection 50 nl of compounds in 1:3 serial dilutions in DMSO (0.5% final DMSO concentration per well) were transferred with an Acoustic Transfer System (ATS) (Biosero) to the cells. Atovaquone (5µM) and puromycin (10 µM) at a single concentration were used as positive controls for the Pbluc and HepG2tox, respectively. 0.5% DMSO was used as negative control for both assays. Pb-Luc sporozoites were freshly dissected from the infected *A. stephensi* salivary glands, filtered twice through a 20 µm nylon net filter (Steriflip, Millipore), counted in a hemocytometer, and adjusted to final concentration of 200 sporozoites per 1 µl in the assay media (DMEM without Phenol Red (Life Technologies, CA), 5% FBS, and 5x Pen Strep Glutamine (Life Technologies, CA). To infect the HepG2-A16-CD81EGFP cells, 1x10**3 sporozoites per well (5 µl) were added with a single tip Bottle Valve liquid handler (GNF), and the plates were spun down at 37 °C for 3 min with a centrifugal force of 330 x g on normal acceleration and brake setting (Eppendorf 5810 R centrifuge). The HepG2-A16-CD81EGFP cell designated for toxicity studies were left uninfected, with 5 µl of additional assay media was added to each well to maintain equal concentrations of compounds with Pb-Luc infected plates. The plates were then incubated at 37 °C for 48 h in 5% CO_2 with high humidity to minimize media evaporation and edge effects.

After incubation at 37 °C for 48 h, the EEF growth and HepG2-A16-CD81EGFP cell viability were assessed by a bioluminescence measurement as follows: Media was removed by spinning the inverted plates at 150 x g for 30 s; 2 µl per well of BrightGlo (Promega) for quantification of Pb-Luc EEFs or CellTiterGlo (Promega) reagent (diluted 1:2 with deionized water) for quantification of HepG2-A16-CD81EGFP cell viability were dispensed with the MicroFlo (BioTek) liquid handler. Immediately after addition of the luminescence reagent, the luminescence was measured by the Envision Multilabel Reader (PerkinElmer).

3.11 *Plasmodium falciparum* stage V gametocytes (UCSD)

This is a modification of the previously reported method (13). Asexual *P. falciparum* parasites (NF54) from a clone G3 were grown at 5% hematocrit in O+ human erythrocytes in serum-containing complete media (RPMI 1640, gentamicin 0.05 mg/ml, hypoxanthine 0.05 mg/ml, HEPES 38.4 mM, sodium bicarbonate 0.2% [w/v], D-glucose 0.2% [w/v], sodium hydroxide 3.4 mM, 11.9% [w/v] heat-inactivated human serum [O+] and at 37 °C under low-oxygen conditions (3% O_2, 5% CO_2, and 92% N_2) and a parasitemia between 0.5% and 3%. Ring-stage parasites were synchronized at 3%

parasitaemia (d-6) with 5% (w/v) D-Sorbitol. On day −4, 45ml of complete media was added to the culture flask to stress via hematocrit drop now at 2.5%. The on d-3 and d-2 only 45 ml of the media was substituted for fresh media. On d-1 a complete media change was performed and the hemocrit restored to 5%. All gametocytes were treated with 50 mM NAG on days 0–9.

Gametocyte stage V parasites were diluted to 0.5%–0.75% gametocytemia and 1.25% hematocrit into serum-free SALSSA screening media (RPMI 1640, gentamicin 0.05 mg/ml, hypoxanthine 0.014 mg/ml, HEPES 38.4 mM, sodium bicarbonate 0.2% [w/v], D-glucose 0.2% [w/v], sodium hydroxide 3.4mM and 0.4% [w/v] AlbuMAX II). Cultures were dispensed (40 µl versus 10 µl) into 384 well plates containing 50 nl or 2.5 nl of compound (final concentration of 1.25 to 12.5 µM) using a MultiFlo dispenser. Plates were incubated at 37°C for 72 h under low-oxygen conditions. For SaLSSA 10 µl (384 well) of 2.5 µM MitoTracker Red CMXRos and 0.13% saponin solution (w/v) in screening media was added to each well, and plates were incubated for 180 min at 37 °C.

Imaging of 384-well plates was performed using a high content imaging system (Operetta, Perkin Elmer) and Harmony software for image analysis. Viability indices were calculated by dividing the particle count of each compound-treated well by the average particle count of the DMSO wells per plate and range from 0 (active compound) to >1 (inactive). Z values were calculated using DMSO-treated gametocytes as positive and uninfected red blood cells as negative wells.

3.12 *Plasmodium falciparum* gamete formation (Imperial)

This is based on methods previously reported (14). Mature *P. falciparum* stage V gametocytes (NF54) were incubated with test compound for 24 h at 37°C in 384-well plates containing complete culture medium (RPMI 1640, 25 mM HEPES, 2 g/l D-glucose, 2 g/l sodium bicarbonate, 50 mg/l hypoxanthine, 10% human serum) under a low oxygen environment (3% O_2/5% CO_2/92% N_2). Gametogenesis was triggered by dispensing 10 µl ookinete medium (RPMI 1640, 25 mM HEPES, 2 g/l D-glucose, 2 g/l sodium bicarbonate, 50 mg/l hypoxanthine, 100 µM xanthurenic acid) also containing 2.7 µg/ml anti-Pfs25-Cy3 into each well and cooling the plate on a metal block at 4 °C for 4 min. Fifteen s later male gametogenesis was recorded by brightfield imaging using times 6 objective and then the plate stored at 26 °C for a further 24 h to allow female gametes to develop. Female gamete formation was assessed by fluorescence microscopy using x6 objective to identify anti-Pfs25-positive cells. Male and female gamete formation was quantified with respect to DMSO and 20 µM gentian violet negative and positive controls.

3.13 Activity against *Cryptosporidium hominis* (CALIBR)

This is based on methods previously reported (10).

4. DMPK (Dundee)

4.1 Aqueous solubility

The aqueous solubility of the test compounds was measured using laser nephelometry, as described previously (15). Compounds were subject to serial dilution from 10 mM to 0.47 mM in DMSO. An aliquot was then mixed with MilliQ water to obtain an aqueous dilution plate with a final concentration range of 250 - 13 µM, with a final DMSO concentration of 2.5 % (v/v). Triplicate aliquots were transferred to a flat bottomed polystyrene plate which was immediately read on the NEPHELOstar (BMG Lab Technologies). The amount of laser scatter caused by insoluble particulates (relative nephelometry units, RNU) was plotted against compound concentration using a segmental regression fit, with the point of inflection being quoted as the compounds aqueous solubility (µM).

4.2 FaSSIF solubility

Drug dissolution in biorelevant media which simulates the liquid environment within the upper gastro-intestinal tract has been shown to have a significant impact on the absorption rate of drugs into the blood stream and is more a more accurate determination of oral dose solubility than simple aqueous solubility performed at neutral pH and without any associated bile acids and fats. This method is based on that of Fagerberg *et al*. (16) where the FaSSIF has pH 6.5 and contains 3 mM sodium taurocholate and 0.75 mM lectithin. FaSSIF blank only has pH 6.5 and is devoid of salts and fat components.

Briefly, approximately 3 mg of test compound is accurately weighed in duplicate and 1 ml of FaSSIF blank or FaSSIF is added to this before vortexing to mix and then incubating in a shaking environment at 1300 rpm at 37 °C for 5 h. The sample is then centrifuged at 10,000 rpm for 3 min before the supernatant is subject to HPLC analysis at 254 nm UV detection wavelength. Typically, 10 µl supernatant injections are sufficient to achieve peak areas which fall within the 6 point standard curve of analyte concentrations injected onto the column, although this may be increased up to 40 µl for more insoluble compounds where necessary.

The amount of drug in the supernatant is interpolated from a 6 point standard curve for the drug prepared over the range 0.05 – 2.5 µg injected onto the column. The HPLC method uses a Luna C18 column (Phenomenex, Macclesfield, UK, 4.6 x 150 mm) with 5 µm particle size. Mobile phase A (MPA) is deionised water containing 0.1% (v/v) formic acid and mobile Phase B (MPB) is MeCN containing 0.1% (v/v) formic acid. The LC system used was a Dionex Ultimate 3000 HLPC where 95% MPA was held for the initial 30 s of the run, then a linear gradient to 5% MPA takes place over the next 4 min before being held at 5% MPA for a further 3.2 min before re-equilibration back to 95% MPA at 9 min. Flow rate was 1 ml/min.

Positive control drugs (cinnarizine and warfarin) are included in every experimental run, to give low and high solubility results for QC purposes.

4.3 CHI logD

Lipophilicity is a key descriptor of the ability of a drug to partition between aqueous and lipid environments within a cell membrane/tissue and hence how easily it could interact with intracellular targets. This can be determined by either log D or Log P measurements, which are traditionally quite time consuming and require quite large amounts of compound to determine accurately.

The CHI Log D assay, first described by by Valkó *et al*. (17), has been adapted and performed at pH 7.4 herein and utilises a 5 µl aliquot from a 10 mM DMSO stock which is diluted to 250 µM by adding 195 µl of MeCN:H_2O (v/v) and mixing in a 96-well plate. An aliquot (10 µl) is then injected onto a Luna C18 column (Phenomenex, Macclesfield, UK, 4.6 x 150 mm) with 5 µm particle size. Mobile phase A (MPA) is 50 mM ammonium acetate (pH 7.4) and mobile Phase B (MPB) is MeCN. The LC system used was a Dionex Ultimate 3000 HLPC where 100% MPA was held for the initial 1.5 min of the run, then a linear gradient to 0% MPA takes place over the next 10 min before being held there for a further 2.5 min before re-equilibration back to 100% MPA at 14 min with data collection continuing until 15 min. Flow rate was 1 ml/min with detection at 254 nm UV and ambient temperature.

The retention time (tR) of the unknown drug is interpolated from the slope and intercept created using retention times of standard mixture components containing paracetamol, theophylline, caffeine, benzimidazole, colchicine, carbamazepine, indole, propiophenone, butyrophenone, valerophenone and hepatophenone (prepared at 10 µg/ml in MeCN:water [1/1, by volume]), which covers the CHI log D range from -1 to 4.44 using the following equations;

CHI = (retention time of unknown x slope of line through standard mixture tR) – intercept of the equation drawn through standard mixture tR

CHI Log D = (0.0525 * CHI) -1.467

4.4 Intrinsic Clearance (CLi) experiments

The mouse and human cryopreserved hepatocytes were supplied by Xenotech (batches MC539 na HUE50H). The procedure was carried out as reported previously (15). Test compound (0.5 µM in DMSO) was incubated with female CD1 mouse, male Sprague-Dawley rat or pooled human mixed gender liver microsomes (Xenotech LLC™; final volume of 0.5 mg/ml in 50 mM potassium phosphate buffer, pH 7.4 containing 0.5% [v/v] DMSO) and the reaction initiated by the addition of excess NADPH (8 mg/ml prepared in 50 mM potassium phosphate buffer, pH 7.4). Immediately, at time zero, then at 3, 6, 9, 15 and 30 min an aliquot (50 µl) of the incubation mixture was removed and mixed with acetonitrile (100 µl) containing internal standard to stop the reaction. Internal standard was added to all samples, the samples centrifuged to sediment precipitated protein and the plates then sealed prior to UPLC-MS/MS analysis using a Quattro Premier XE (Waters Corporation, USA). XLfit software (IDBS, Guildford UK) was used to calculate the exponential decay and consequently the rate constant (ke^{-1}) from the ratio of peak area of test compound to internal standard at each time point. The rate of intrinsic clearance (CLi) of each test compound was then calculated using the following calculation:

CLi (ml/min/g liver) = ke^{-1} x V x Microsomal protein yield

where V (ml/mg protein) is the incubation volume/mg protein added (0.5) and microsomal protein yield is taken as being 52.5 mg protein per g liver. Verapamil (0.5 µM) was used as a positive control to confirm acceptable assay performance. Experiments were performed using a single time-course experiment.

4.5 Hepatocyte stability

Cryopreserved hepatocytes were used to take non-CYP mediated Phase I and Phase II enzymes into account in the determination: hence values for intrinsic clearance using whole cells tend to be higher than those determined using liver microsomes or other subcellular liver fractions and could be more predictive of a metabolic rate determined *in vivo*.

Cryopreserved mouse (batch MC539) and human (batch HUE50H) hepatocytes were supplied by ThermoFisher Scientific, Paisley, UK and Xenotech, LLC, respectively. Cryopreserved hepatocytes were rapidly thawed in a pre-heated water bath at 37 °C before adding to 50 ml of pre-warmed CHRM media (ThermoFisher Scientific, Paisley, UK) and mixed gently. Hepatocytes were then centrifuged at room temperature at 55 x g for 3 min or 100 x grcf for 10 min for mouse and human respectively. before re-suspending in 1 ml Williams medium E (WME) supplemented containing media cocktail and dexamethasone both supplied by ThermoFisher Scientific)The supplemented WME was pre-warmed to 37 °C and had been bubbled through with CO_2 for at least 30 minutes prior to use. Cell viability and density were determined by trypan blue exclusion (10% trypan blue, by volume) using a disposable haemocytometer before being diluted to a working cell density of 0.5 million viable cells/ml in supplemented WME. Only suspensions which achieved >80% post-thaw viability were used in experiments.

Test compounds were supplied as 10 mM DMSO stock solutions and were diluted in supplemented WME in a two stage dilution to achieve a final stock concentration of 1 µM containing 1% DMSO (v/v). 200 µl of these solutions were transferred to a clean 48-well non-collagen coated plate and pre-warmed in a humidified 5% CO_2 atmosphere incubator and subject to gentle mixing (~90 rpm) on a orbital rotating platform. The reactions were initiated by the addition of 200 µl of hepatocyte suspension and 20 µl sample was removed at 10 time points up to 120 min and terminated by precipitation in 80 µl MeCN (containing internal standard) in a 96 well polycarbonate analysis block. Hence samples were incubated at final substrate concentration of 0.5 µM containing 0.5% (v/v) DMSO and 0.25 million cells/ml. The solvent component was reduced to 50% (by volume) in terminated samples by adding 100 µL H_2O:MeCN before the sample plate was centrifuged at 3570 rpm for 10 min to pellet the cellular debris and the supernatants transferred to a clean 96 well plate prior to analysis using identical methods, equipment and data processing as for microsomal CLi determinations above except that a scaling factor of 120 million cells/g liver was applied to the rates of reaction to express the values as ml/min/g liver.

Positive control incubations with 7-ethoxycourmarin, 7-hydroxycoumarin, verapamil and phthalazine were included in all experiments to confirm that cytochrome P450, Glucuronidation/sulfation, and

aldehyde oxidase pathways were active and were able to contribute to the metabolic rate, provided that the compounds tested were substrates for these enzymes.

4.7 Fluorescence-based CYP Inhibition using recombinantly expressed CYP bactosomes

Fluorogenic CYP inhibition studies were conducted at 37 °C in 96-well, flat-bottom, clear polystyrene plates. Incubation mixtures containing EasyCYP bactosomes (1000 pmol/ml, 10 mg/ml Cypex ™, Dundee, UK), fluorogenic substrate (Cypex ™) and 50 mM potassium phosphate buffer (pH 7.4) were prepared at the following final concentrations: CYP1A2, 5 pmol/ml + 35 µM ethoxyresorufin (ER); CYP2C9, 10 pmol/ml + 30 µM 7-methoxy-4-(trifluoromethyl)-coumarin (MFC); CYP2C19, 5 pmol/ml + 25 µM 3-cyano-7-ethoxycoumarin (CEC); CYP2D6, 10 pmol/ml + 6 µM 7-methoxy-4-(aminomethyl)-coumarin (MAMC); CYP3A4, 10 pmol/ml + 1 µM diethoxyfluorescein (DEF) and 10 pmol/ml + 15 µM 7-benzyloxyquinoline (BQ). Bactosome control protein was included in reactions to give a final concentration of 0.0125 (1A2 and 2C9 only) or 0.025 mg/ml (all other isoforms). Test compounds were prepared as 0.5 mM solutions in DMSO and serially diluted 1 in 3.03, 1 in 3.3 alternatively in a v-bottomed 96 well plate to give a 7 point concentration range of 500 – 50 µM. Positive control inhibitor, miconazole, was prepared as a 500 µM solution in DMSO and similarly diluted.

For each isoform, 220 µl of incubation mix was added to each well of a 96-well, flat-bottom, clear polystyrene plate. Aliquots (5 µl) of each compound **5** concentration was then mixed with 220 µl of the incubation mix and pre-incubated at 37 ˚C for 5 min (final test compound concentration range: 10, 3.3, 1.0, 0.33, 0.1, 0.033, 0.01 and 0 µM; miconazole 10, 3.3, 1.0, 0.33, 0.1, 0.033, 0.01 and 0 µM) both containing final DMSO concentrations of 2% (v/v). Reactions were initiated by the addition of 25 µl regenerating cofactor solution (28 mM glucose-6-phosphate, 2.2 mM NADP, 6 U/ml glucose-6-phosphate dehydrogenase (from Baker's yeast *S. cerevisiae*) in 2% w/v $NaHCO_3$, Sigma) and subsequent production of fluorescence metabolite measured at 1 min intervals over a 10 min period using a BMG Optima fluorescence detector (ER: Exc 540nm, Em 590nm, MFC: Exc 430 nm, Em 540nm, CEC: Exc 405 nm, Em 450 nm, MAMC: Exc 405 nm, Em 450 nm, DEF: Exc 430 nm, Em 540 nm, 7BQ: Exc 485 nm, Em 520 nm). Reaction times were verified to be within the limits of kinetics linearity.

Fluorescence responses were calculated as a percentage of uninhibited solvent matched control and plotted against compound concentration to provide IC_{50} values using the following equation using XLfit curve fitting software (IDBS) ensuring that the control values fell within 80-120% of mean replicate wells across the plate.

4. 8 Human ether-à-go-go related gene (hERG) K^+ assay (Outsourced)

Compounds were tested for inhibition of the human ether-à-go-go-related gene (hERG) K^+ channel using IonWorks patch clamp electrophysiology. Eight-point concentration-response curves were generated on 2 occasions using 3-fold serial dilutions from the maximum final assay concentration.

5. *In vivo* pharmacokinetic and efficacy studies

5.1 *In vivo* pharmacokinetics (Dundee)

Compound **5** was dosed as a bolus solution intravenously at 3mg free base/kg (dose volume: 5 ml/kg; dose vehicle: 10% DMSO, 40% PEG400 and 50% MilliQ water) to female Balb/c mice (n=3) or dosed orally by gavage as a solution at 10 mg free base/kg (dose volume: 10 ml/kg; Dose vehicle: 0.5% hydroxypropylmethylcellulose, 0.4% Tween 80 and 0.5 benzyl alcohol to female BALB/c mice (n=3/dose level). Blood samples (10 µl) were taken from each mouse at 5, 15 and 30 min, 1, 2, 4, 6, 8 and 24 h post-dose, mixed with nine volumes of distilled water and stored frozen until UPLC-MS/MS analysis. The level of each compound in mouse blood was determined by UPLC-MS/MS as previously reported (18). Pharmacokinetic parameters were derived from the blood concentration time curve using PKsolutions software v 2.0 (Summit Research Services, USA).

5.2 *In vivo* antimalarial efficacy studies in *P. falciparum* (SwissTPH)

Compounds were tested in the murine *P. falciparum* SCID model essentially as described (19). Briefly, compound **5**, formulated in 0.5% hydroxypropylmethylcellulose (HPMC); 0.4% Tween 80; 0.5% benzoyl alcohol, was administered to a cohort of age-matched female immunodeficient NOD-*scid IL-2Rγ*^{null} mice (The Jackson Laboratory, Bar Harbor, ME) previously engrafted with human erythrocytes (generously provided by the Blood Bank in Zürich, Switzerland). The mice were intravenously infected with 2×10^7 *P. falciparum* Pf3D70087/N9-infected erythrocytes (day 0). On day 3 after infection, mice (n=2) were randomly allocated to treatments that were administered once a day for 4 consecutive days by oral gavage at 10 ml/kg. Parasitemia was measured by microscopy and flow cytometry using anti-murine erythrocyte TER119 monoclonal antibody (Pharmingen, San Diego, CA) in serial 2 µl blood samples taken every 24 h until assay completion.

Serial samples of peripheral blood (25 µl) were taken from the mice of the efficacy experiment by tail puncture at 1, 2, 6 and 24 h after the first administration. The samples were immediately lysed by mixing with 25 µl of water, immediately frozen on dry ice and stored at -80°C until bioanalysis (carried out at GSK, Tres Cantos). The compounds were extracted from 10 µl of each lysate with 180 µl AcN:MeOH (80:20; v:v) and stored frozen at -80º C until analysis by LC/MS/MS in AB Sciex API4000 (AB Sciex, Framingham, MA). The compound concentration versus time data were analyzed by non-compartmental analysis (NCA) using PhoenixR Version 6.3 (Pharsight Corporation, Mountain View, CA, USA). Additional statistical analysis was performed with GraphPad PrismR (GraphPad Software Inc, San Diego CA, USA).

5.3 *In vivo* antimalarial efficacy studies in *P. falciparum* (TAD)

The efficacy of compound **5** against *P. falciparum in vivo* was evaluated in a non-myelodepleted humanized mouse model (20). Female immunodeficient NOD-*scid IL-2Rγ*^{null} mice (NSG) (Charles River, France) of 25-28 gr bodyweight were housed in The Art of Discovery animal facility at BIC Bizkaia building (Derio, Basque Country, Spain), which is equipped with HEPA filtered in/out air-conditioned with 15 air renovations per h at 22 ± 2 ºC; 40-70% relative humidity; 12 h light/dark period. The mice were accommodated in racks with ventilated disposable cages (Innovive) in groups

of up to five individuals with autoclaved dust-free corncob bedding (Innovive) and fed with γ-irradiated standard pellet (Envigo) and ultra-filtered water (Innovive) *ad libitum*.

The mice of the study were engrafted by daily intraperitoneal injection of 1 ml of human erythrocytes (Basque Center of Transfusion and Human Tissues, Galdakao, Spain and Bank of Blood and Tissues, Barcelona, Spain) suspended in RPMI1640 medium, 25% (vol/vol) decomplemented human serum, 3.1 mM hypoxanthine at 50%-75% hematocrit. The suspension was injected daily during the whole experiment. At a minimum of 40 % of chimerism (7-10 days after start of chimerization), the mice were infected by i.v. route with 35×10^6 *Plasmodium falciparum* Pf3D7$^{0087/N9}$-infected erythrocytes suspended in 0.3 ml of saline. The *P. falciparum* Pf3D7$^{0087/N9}$ strain (19) was kindly donated by Dr. Sergio Wittlin (Swiss Tropical and Public Health Institute, Basel, Switzerland). The inoculum was obtained from blood of CO_2-euthanized donor mice harbouring 5-10% parasitemia. A single inoculum was used for all individuals of the efficacy study.

The drug was daily formulated as a transparent solution in 0.5% hydroxypropylmethylcellulose, 0.4% Tween 80, 0.5% benzyl alcohol in ddH_2O (HPMC). Drug treatment was administered once a day by oral gavage with 20G straight, reusable, feeding needles (Fine Science Tools GmbH) at 10 ml/kg for four consecutive days starting 72h after infection (~1% of patent parasitemia in peripheral blood).

Serial 2 µl blood samples of peripheral blood from *P. falciparum*-infected mice were stained with TER-119-Phycoerythrine (marker of murine erythrocytes) and SYTO-16 (nucleic acid dye) and then analyzed by flow cytometry (Attune NxT Acoustic Focusing Flow Cytometer, InvitroGen) as described (21). The limit of quantitation is set to 0.01% for a minimum of 100 infected events as statistically significant sample ($\sim10^6$ total erythrocytes counted). Parasitemia is expressed as the % of parasitized erythrocytes with respect the total erythrocytes in circulation and/or as the absolute concentration of circulating parasitized erythrocytes. A qualitative analysis of the effect of treatment on *P. falciparum* Pf3D7$^{0087/N9}$ was assessed by microscopic analysis of Giemsa-stained blood smears prepared with blood samples at 48 h and 96 h after drug treatment inception.

The concentration of compound **5** in whole blood of the mice of the efficacy study was measured in 25 µl samples of peripheral blood taken at 0.5, 1, 2, 4, 8, 23, 71, 74 and 95 h after the first dosing. The samples were mixed with 25 µl of H_2O MilliQ, immediately frozen on dry ice and stored at -80 ºC until analysis. Blood from control *P. falciparum*-infected humanized mice was used for preparation of standard curves, calibration and quality control purposes. The compound was extracted from 10 µl of lysates obtained by protein precipitation of diluted blood samples using standard liquid-liquid extraction methods. The samples were analyzed by LC-MS/MS for quantitation in a Waters UPLC-TQD (Micromass, Manchester, UK). Blood concentration *vs* time was analyzed by non-compartmental analysis (NCA) using Phoenix WinNonlin vers.7.0 (Certara), from which exposure-related values (C_{max} and AUC_{0-t}) and t_{max}, were estimated.

Efficacy is expressed as the effective dose in mg/kg and the corresponding average daily exposure that reduced parasitemia at day 5 of the assay by 90 % with respect to untreated mice (denoted as ED_{90} and AUC_{ED90}, respectively). These parameters are calculated by fitting the variables X= $\log_{10}$[dose

level in mg/kg] and X= $\log_{10}$[AUC of compound during the first 23 h after the first drug administration, in ng·h·ml^{-1}], respectively, *versus* Y= $\log_{10}$[parasitemia at day 5 of the assay], where each individual of the study is defined as an ordered pair, to the function **Y=Bottom + (Top-Bottom)/(1+10$^{((LogED50-X)*HillSlope)}$)**.

Data analysis was performed using GraphPad Prism 7.0 (GraphPad Software), Excel 2016 (Microsoft) and R free software (*https://www.r-project.org*) for data plotting and statistical modelling. Phoenix WinNonlin vers.7.0 (Certara) was used for PK Non-Compartmental Analysis.

5.4 Measurement of anti-Cryptosporidium *in vivo* efficacy in IFN-γ-knockout mice (Georgia)

The Nluc-*C. parvum* IFN-γ-knockout (IFN-γ KO) mouse *in vivo* experiments were run in accordance with the published method (22). Briefly, female C57BL/6 IFN-γ KO mice, aged 6–8 weeks were infected with 10,000 *C. parvum* UGA1 Nluc (Vinayak et al 2015, Nature) transgenic oocysts. Mice (n=5 per group) were administered 20 mg/kg of compound **5** formulated in 3% ethanol, 7% Tween 80, 90% saline or the formulation (vehicle) by oral gavage. Treatment with compound **5** (20 mg/kg) or vehicle was started at day 4 post infection and continued daily for 7 days (day 4 to day 10). Fecal samples from mice cage were collected (during and after treatment, till day 35), and luminescence assays were performed as described previously (22, 23). For the Nluc assay, 20 mg of mashed fecal material was suspended in 1 ml of lysis buffer (50mM TrisHCl pH7.6, 2mM DTT, 2mM EDTA, 10% glycerol, 1% triton-X 100) and 3 mm glass beads were added to the tube followed by vortexing for 1 min. The lysate was subjected to a brief spin in the centrifuge, and the supernatant (three aliquots of 100 µl each) were added to 96 well white plates. 100 µl of reconstituted NanoGlo buffer containing 1:50 of NanoGlo substrate (Promega) was added to each well, and luminescence was read on a Synergy H4 Hybrid Microplate Reader (BioTek Instruments).

5.5 Measurement of anti-Cryptosporidium *in vivo* efficacy in NOD SCID gamma mice (Vermont)

The cryptosporidiosis NOD SCID gamma mouse model were run in accordance with the method published in R. S. Jumani *et. al.* (10). Three to four week old male NOD SCID gamma mice (NOD.C*g-Prkdc^{scid} Il2rg^{tm1Wjl}/SzJ*) were purchased from Jackson Laboratory (Bar Harbor, ME) and were housed for one week prior to infection with ~10^5 *C. parvum* Iowa isolate oocysts by oral gavage. Compounds were prepared for dosing by suspension in DMSO, sonication and freezing aliquots at -80 °C. On each day of treatment, aliquots of compound were thawed, mixed well by vortexing, diluted in 1% hydroxypropylmethylcellulose (HPMC) (5% DMSO in 100 µl 1% HPMC), sonicated, mixed thoroughly, and then administered by oral gavage. Oral treatment with experimental compounds, paromomycin (positive control), or the HPMC/DMSO vehicle alone was begun 7 days after infection, and continued for 7 days. Fecal oocyst shedding was quantified by qPCR just before starting treatment and one day after completion of treatment (10).

6. Modelling and Molecular Dynamics Simulations

6.1 Initial models

The crystal structure of *Pf*KRS1 dimer in complex with compound **2** (PDB 6AGT) was used to construct all *Pf*KRS1 models for molecular dynamics (MD) simulations: with a bound ligand (compound **5**), in the presence and absence of lysine. A model of the human KRS in complex with compound **5** was obtained by superimposing the *Pf*KRS1 structure with the available crystal structure of the human KRS in complex with cladosporin (PDB ID 4YCU) (24). Finally, the crystal structure of *Cp*KRS with a bound compound **5** was simulated in the presence and absence of lysine. *Pf*KRS1, *Cp*KRS and *Hs*KRS were also simulated in the apo state (no ligand but in the presence of lysine). A summary of models and MD simulations performed in this article is presented in Table S4

6.2 System preparation.

The systems for MD simulations were prepared with the utility LEaP, which is integrated in the suite of programs AMBER 16 (25). The ff14SB force field (26) was used. The N- and C-termini of the proteins were capped with an acetyl (ACE) and methylated amino group (NME), respectively. The geometries of the ligands were refined with Gaussian03 (27) at the HF/6-31G* level. The optimized geometries were used to calculate the electrostatic potential-derived (ESP) charges using the RESP methodology (28), as implemented in the Antechamber module in AMBER 16. The force field parameters for the ligand were generated with the Antechamber module, using the general AMBER force field (GAFF 2.0). The force field parameters and partial charges for the isolated lysine were taken from a study by Horn (29). Each simulated system was immersed in a water box (TIP3P water model) and neutralized by adding the appropriate number of counterions. This was followed by steepest-descent energy minimization to remove steric clashes.

6.3 MD simulation protocol.

MD simulations were performed using the pmemd.cuda module of AMBER 16. The cut-off distance for the non-bonded interactions was set to 10 Å. The periodic boundary conditions were used. Electrostatic interactions were treated using the smooth particle mesh Ewald method (30). The SHAKE algorithm was applied to all bonds involving hydrogen atoms, and a time step of 2 fs was used throughout (31). Each energy minimized system was heated to 300K, equilibrated for 10 ns, and further simulated for 500 ns without any restraints.

6.4 MD Analysis.

Protein structures and MD trajectories were visually inspected and analyzed using the molecular visualization programs PyMOL (32) and VMD (33). Both copies of the dimer systems were used for analysis. Interatomic distances, angles and root-mean-square deviations (RMSD) with respect to a reference structure were monitored using the *CP*PTRAJ module in AmberTools (25). The ligand-*Pf*KRS1 interaction diagram was prepared using Maestro 11.1 software package (34). Energy decomposition analysis was performed using the MMGBSA method as implemented in the MMPBSA.py program (35).

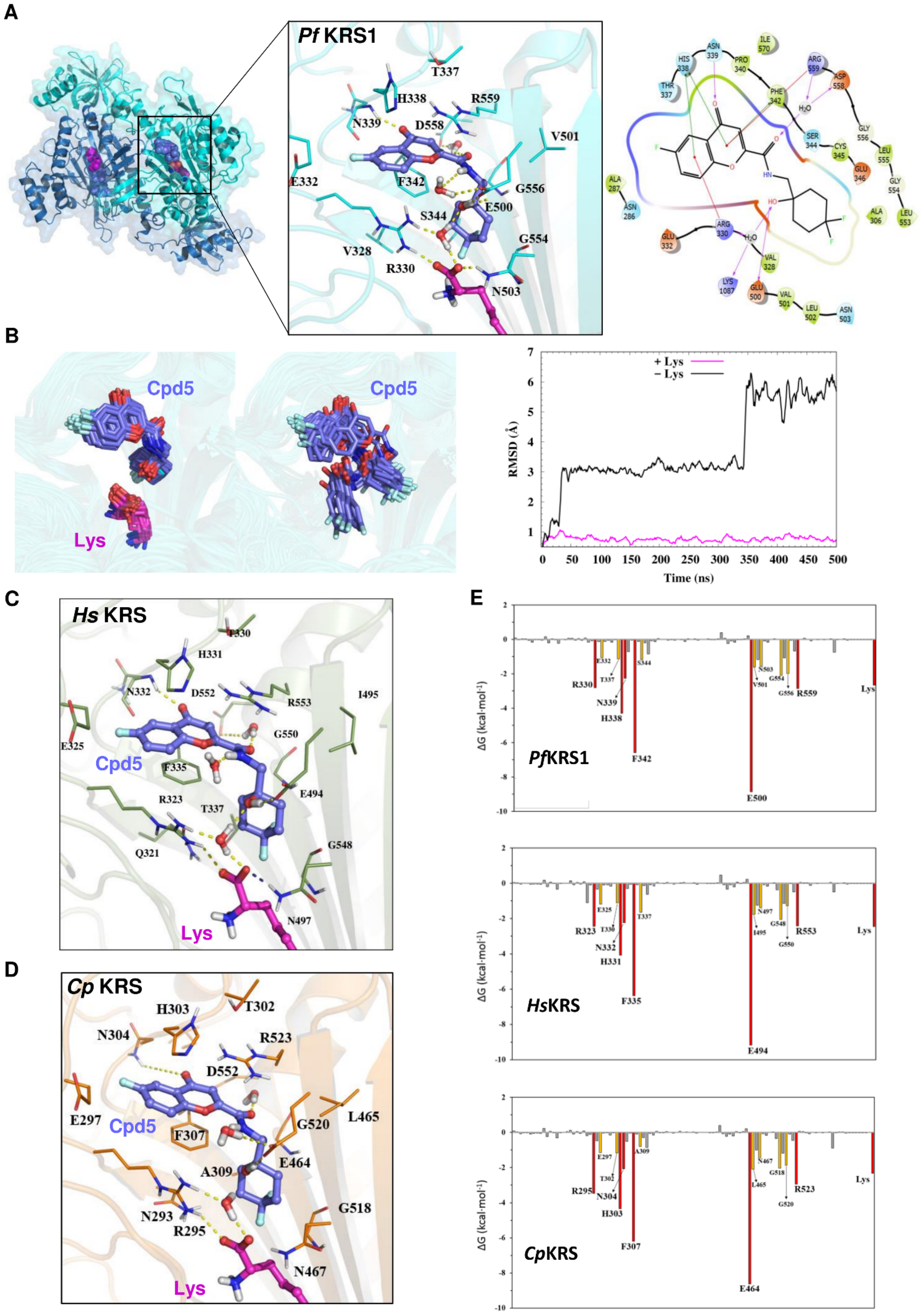
A
Pf KRS1
T337
H338
R559
N339
D558
V501
E332
F342
G556
E500
S344
G554
V328
R330
N503
B
Cpd5
Lys
Cpd5
+ Lys
− Lys
RMSD (Å)
Time (ns)
C
Hs KRS
T330
H331
N332
D552
R553
I495
E325
G550
Cpd5
F335
E494
R323
T337
Q321
G548
N497
Lys
D
Cp KRS
H303
T302
N304
R523
D552
E297
Cpd5
F307
G520
L465
A309
E464
N293
R295
G518
N467
Lys
E
ΔG (kcal·mol⁻¹)
PfKRS1
R330
E332
T337
N339
H338
S344
F342
E500
V501
N503
G554
G556
R559
Lys
HsKRS
R323
E325
T330
N332
H331
T337
F335
E494
I495
N497
G548
G550
R553
CpKRS
R295
E297
T302
N304
H303
A309
F307
E464
L465
N467
G518
G520
R523

Figure S5: Modelling of *Pf*KRS1, *Hs*KRS and *Cp*KRS in complex with compound **5**.

(A) left: Overall structure of the dimeric *Pf*KRS1 system and structural details of the *Pf*KRS1 active site (cyan), in complex with compound **5** (violet) and lysine (magenta); *right*: Ligand interaction diagram for compound **5** bound in the active site of *Pf*KRS1. (B) *left*: Representative snapshots of compound **5** (violet) bound to *Pf*KRS1 active site (transparent cyan) displaying 30 conformations from the MD simulations in the presence and absence of lysine (magenta); *right*: RMSD of compound **5** along the MD simulations of *Pf*KRS1 in the presence and absence of lysine. (C) Interactions of compound **5** (violet) in the active site of *Hs*KRS (green) in the presence of lysine (magenta). (D) Interactions of compound **5** (violet) in the active site of *Cp*KRS (orange) in the presence of lysine (magenta). (E) Per-residue contributions to the binding free energy of compound **5** to *Pf*KRS1 (*top*), *Hs*KRS (*middle*) and *Cp*KRS (*bottom*). The decomposition analysis of the binding free energy was performed using MM-GBSA (see details in Methods section).

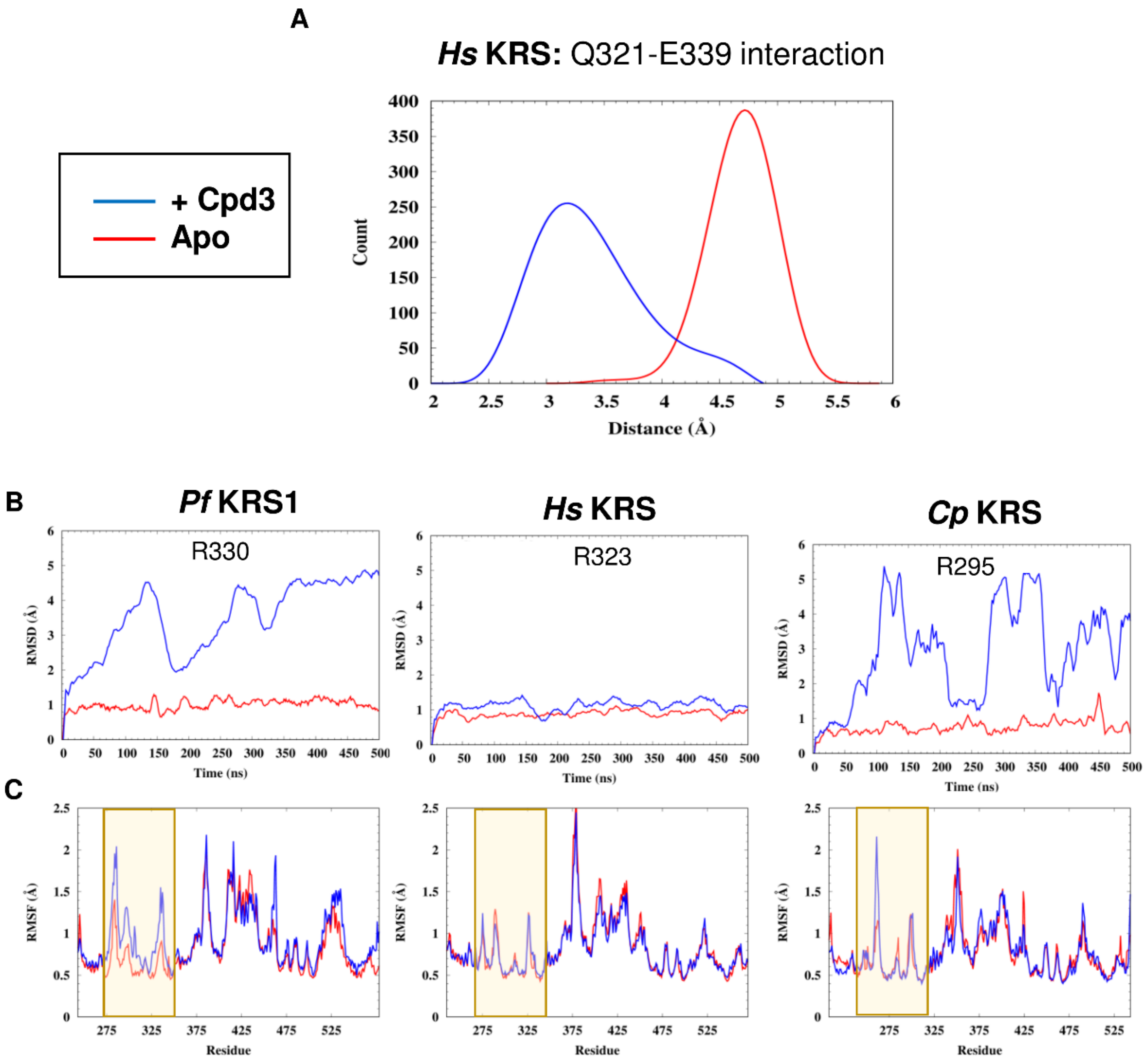

Figure S6: Differential flexibility of residues near the active site in *Pf*KRS1, *Hs*KRS and *Cp*KRS .
(A) Distributions from the MD simulations of the distance between Q321 and E339 sidechains in apo (blue) and ligand-bound *Hs*KRS (red). (B) RMSD evolution of *Pf*KRS1 R330 (*left*), *Hs*KRS R323 (*middle*) and *Cp*KRS R295 (*right*) along the MD simulations in the apo (blue) and ligand-bound (red) states. (C) Root mean square fluctuations (RMSF) of individual residues in MD simulations of *Pf*KRS1 (*left*), *Hs*KRS (*middle*) and *Cp*KRS (*right*) in the apo (blue) and ligand-bound (red) states

Table S4. Summary of MD simulations.

System	Simulation time (ns)
apo-KRS	
***apo-Pf*KRS1**	500
***apo-Hs*KRS**	500
***apo-Cp*KRS**	500
KRS in complex with cpd ***5***	
***Pf*KRS1**	500
***Hs*KRS**	500
***Cp*KRS**	500
***Pf*KRS1(-Lys)**	500

All the systems were modelled as KRS dimers. All the systems, except *Pf*KRS1(-Lys), also have a bound lysine.

7. X-ray Crystallography

7.1 *Pf*KRS1 complexes (New Delhi, Dundee)

Protein:ligand complexes *Pf*KRS1 were obtained by co-crystallisation using methods described previously (36).

Briefly *Pf*KRS1 was prepared at 13.5 mg/ml in 50 mM Tris–HCl pH 8.0, 200 mM NaCl, 10 mM 2-mercaptoethanol. Ligands were prepared at 20mM stock concentration in 100% DMSO before addition to protein solution to a final concentration of 400uM. Crystallisation was carried by hanging drop vapour diffusion preparing 1µl:1µl drops of protein:reservoir. The reservoir solution consisted of 0.1 M Bis-Tris pH 6.5, 2 % (v/v) Tascimate pH 6.0, 20 % (w/v) PEG 3350. Plate shaped crystals were obtained after 2-4 weeks incubation at 277 K.

Protein crystals were cryoprotected in mother liquor plus 20% glycerol before freezing in liquid nitrogen in preparation for data measurement. Diffraction data were measured at the European Synchrotron Radiation Facility (ESRF) beamlines BM14 (*Pf*KRS1;**2**), ID-29 (*Pf*KRS1:**5**) and using a Rigaku Micromax-007 rotating anode source coupled with a Saturn 944 CCD detector (*Pf*KRS:**4**). Data for *Pf*KRS:**2** were integrated, reduced and scaled using HKL2000 (37) with phasing carried out by molecular replacement with Phaser (38) as implemented in Phenix (39) using *Pf*KRS1:lys (PDB 4H02)

as a search model. The structure was refined using Phenix. Data for *Pf*KRS1:**4** and *Pf*KRS1:**5** were integrated with XDS (40) and reduced and scaled using AIMLESS (41). Phasing was carried out by molecular replacement using MOLREP using PfKRS1:lys (PDB 4H02) as a search model. The structure was refined using REFMAC5 (42) from the CCCP4 suite of programmes (43). For all *Pf*KRS1 complexes manual model alteration was carried out using Coot (44).

*7.2 Cp*KRS complexes (Beryllium Discovery, SSGCID, Dundee)

To obtain crystals of *Cp*KRS:lys and *Cp*KRS:lys:cladosporin, *Cp*KRS was concentrated to 35 mg/ml and incubated with 3 mM $MgCl_2$, L-lysine, and AMPPNP, then mixed 1:1 with a solution containing 25% (w/v) PEG-3350, 0.2 M lithium sulfate, 0.1 M Tris base/ HCl, pH 8.5. Crystals were then grown at 289 K by sitting drop vapor diffusion with 0.4 µl of protein/ligand complex mixed with 0.4 µl reservoir solution. Crystals were harvested with 20% ethylene glycol and flash frozen. For *Cp*KRS bound to cladosporin and lysine, crystals from the same well were soaked overnight in a solution that is 70% of the previous condition, 20% ethylene glycol, 10% DMSO, with 2 mM cladosporin for 24 h, then harvested and flash frozen. Crystals of *Cp*KRS:**5** were also obtained by soaking methods. *Cp*KRS protein was prepared at 35 mg/ml and incubated with 1 mM L-lysine prior to crystallisation by sitting drop vapour diffusion. Drops were prepared mixing 1ul protein with 1 µl reservoir solution consisting of 25% (w/v) PEG-3350, 0.2 M lithium sulfate, 0.1 M Tris base/ HCl, pH 8.5. Crystals were transferred to soaking solution consisting of reservoir solution supplemented with 2 mM **5** prepared from a stock solution of 200 mM in DMSO and incubated overnight at 291K. Crystals were passed through a cryprotective solution of reservoir solution supplemented with 20% ethylene glycol before flash freezing in liquid nitrogen in preparation for data measurement.

Data for *Cp*KRS:lys and *Cp*KRS:lys:cladosporin were measured beamline 21-ID-F at the Advanced Photon Source (APS). Data for *Cp*KRS:**5** was measured at beamline I24 at Diamond Light Source (DLS)

Diffraction data for *Cp*KRS:lys and *Cp*KRS:lys:cladosporin data were processed with XDS and XSCALE (40), and data for *Cp*KRS:**5** processed with XDS and xia2 (45). The *Cp*KRS:lys structure was determined by molecular replacement with Phaser (46) using 3BJU as a starting model. Lysine-bound *Cp*KRS was then used as the model to determine the structure of *Cp*KRS:lys:cladosprin. Structure refinement of *Cp*KRS:lys and *Cp*KRS:lys:cladosporin was carried out using Phenix (39) and manual model building with Coot (44). The structure of *Cp*KRS:**5** was refined using REFMAC5 (42) with manual alteration of all complexes carried out using Coot (44). The quality of all *Cp*KRS structures was validated with Molprobity (47).

Data measurement and refinement statistics are shown in Table S5

	*Pf*KRS1:Lys:2	*Pf*KRS1:5	*Pf*KRS1:4	*Cp*KRS:Lys	*Cp*KRS:Lys:Clado	*Cp*KRS:Lys:5
**PDB code**	6AGT	6HCU	6HCV	5ELN	5ELO	6HCW
	Data Measurement					
**Source**	ESRF BM14	ESRF ID29	Rigaku 007	APS 21-ID-F	APS 21-ID-F	DLS I24
**Space Group**	P1	$P2_12_12_1$	$P2_12_12_1$	$P2_1$	$P2_1$	$P22_12_1$
**Unit Cell Dimensions**						
**a,b,c(Å)**	72.8,104.3 100.6	73.2, 95.3, 166.5	73.2,95.3,166.5	72.9,119.5,143.6	73.2, 120.7, 143.3	73.0, 116.5, 142.7
**α,β,γ (°)**	89.9, 69.6, 61.1	90.0, 90.0, 90.0	90.0,90.0, 90.0	90.0, 90.3, 90.0	90, 90.2, 90	90.0, 90.0, 90.0
**Resolution (Å)**	50-1.95 (1.98-1.95)	82.75 -1.62 (1.66-1.62)	47.73-2.20 (2.26-2.20)	50.0–1.90 (1.95–1.90)	50.0–1.90 (1.95–1.90)	72.94 – 1.46 (1.50-1.46)
**Observations**	725596	636756	286641	734082	803555	1143460
**Unique Observations**	166896	148069	59459	192804	194875	205432
**Rmerge (%)**	8.5 (68.5)	6.2 (87.2)	11.7 (38.4)	9.5 (55.3)	10.5 (58.7)	7.6 (65.9)
**CC ½ high res**	0.922	0.59	0.49	0.83	0.70	0.56
**I/σI**	21.7 (2.4)	10 (1.7)	9 (2.3)	12 (2.8)	11 (2.8)	10 (1.4)
**Completeness (%)**	96.9 (96.8)	99.9 (100)	98.7 (90.3)	99.7 (99.9)	99.6 (99.9)	97.6 (80.5)
**Redundancy**	4.3 (4.4)	4.3 (4.4)	4.8 (2.4)	3.80 (3.80)	4.1 (4.2)	5.6 (2.6)
	Refinement Statistics					
**Resolution Range (Å)**	32-1.9	82.75 -1.62	47.73-2.20	19.98–1.90	47.07–1.90	72.94 – 1.46
**R-factor (R_{work}/R_{free})**	20.3/24.3	18.3/22.6	21.3/25.4	15.4/19.2	20.4/24.6	18.7/21.5
**Number of atoms [a]**	15831/70/84/571	8005/50/47/966	7792/46/-/539	15969/-/87/1942	15928/84/34/1969	8029/28/50/907
**Mean B-factor (Å^2) [a]**	45/40/32/38	26/17/38/37	29/20/-/32	26/-/31/33	30/18/83/35	15/10/10/25
**RMS bond length deviation (Å)**	0.08	0.02	0.01	0.008	0.01	0.02
**RMS bond angle deviation (°)**	1.16	2.24	1.30	0.89	1.07	1.92

Table S5. Data measurement and refinement statistics for KRS complexes.

Data measurement statistics for the highest resolution shell are shown in parenthesis. [a] Number of atoms and mean B-factors are shown for protein/heteroatom/ligand/water respectively

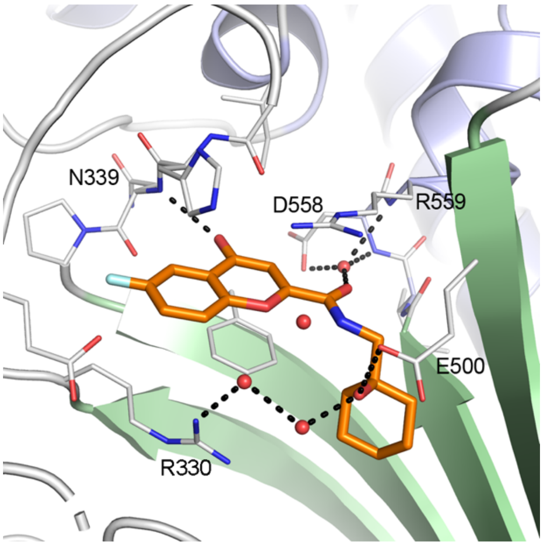

Figure S7: Binding mode of compound **4** with *Pf*KRS1

Binding mode of **4** (C atoms gold) bound to *Pf*KRS1. H-bonds are shown as dashed lines and key residues labelled for clarity

8. Ethical Use of Animals and Human Tissue

All NOD SCID gamma mouse studies were performed in compliance with animal care guidelines and were approved by the University of Vermont Institutional Animal Care and Use Committee. The University of Vermont is fully accredited by AAALAC (Animal Welfare Assurance Number: D16-00193 (A3301-01)).

The anti-cryptosporidium IFN-γ-knockout mouse studies described in this paper were approved by the Institutional Animal Care and Use Committee of the University of Georgia (animal use protocol number A2016 01-028-Y1-A4).

Animal experiments performed at The Art of Discovery were approved by The Art of Discovery Institutional Animal Care and Use Committee (TAD-IACUC). This Committee is certified by the Biscay County Government (Bizkaiko Foru Aldundia, Basque Country, Spain) to evaluate animal research projects from Spanish institutions according to point 43.3 from Royal Decree 53/2013, from the 1st of February (BOE-A-2013-1337). All experiments were carried out in accordance with European Directive 2010/63/EU.

The animal experiments described here are carried out at the Swiss Tropical and Public Health Institute (Basel, Switzerland), adhering to local and national regulations of laboratory animal welfare in Switzerland (awarded permission no. 2303). Protocols are regularly reviewed and revised following approval by the local authority (Veterinäramt Basel Stadt).

All regulated procedures on living animals in the Drug Discovery Unit, University of Dundee were carried out under the authority of project licence(s) issued by the Home Office under the Animals

(Scientific Procedures) Act 1986, as amended in 2012 (and in compliance with EU Directive EU/2010/63). Licence applications have been approved by the University's Welfare and Ethical Use of Animals committee, before submission to the Home Office. The committee has a general remit to develop and oversee policy on all aspects of the use of animals on University premises and is a sub-committee of the University Court, its highest governing body.

Human erythrocytes were obtained with ethical approval from national blood transfusion services. Human microsomes and hepatocytes were obtained from commercial sources. Human ileocecal adenocarcinoma cells obtained from ATCC.

Author Contributions

Author	Institution	Role
Mark Anderson	University of Dundee	Conducted and analyzed data for in vitro evaluation of *P. falciparum* blood stage activity
Iñigo Angulo-Barturen	TAD	Design, execution, and analysis of *in vivo* efficacy of compound **5** in the *P. falciparum* mouse model.
Beatriz Baragaña	University of Dundee	*in silico* drug design, interpretation of biological activity data, *in vitro* DMPK and *in vivo* PK and efficacy data, iterative drug design, chemical synthesis, project management, writing paper
Jake Baum	Imperial College	Oversaw the transmission blocking assays at Imperial College.
Carrie F. Brooks	University of Georgia	Assisted in in vivo efficacy studies of compound **5** in the Nluc cryptosporidiosis INF-γ -knockout mouse model
Juan A. Bueren-Calabuig	University of Dundee	Molecular dynamics (MD) simulations, interpretation of data from MD and writing paper
Ryan Choi	University of Washington	Conducted and analyzed data for *in vitro* evaluation of inhibition of *Pf*, *Cp* and *Hs*KRS and thermal shift experiments. Edited manuscript.
Caitlin A. Cooper	University of Georgia	Carried out the *P. vivax* liver stage assays
Sebastian Damerow	University of Dundee	Development of biochemical assays
Michael Delves	Imperial College	Coordinated and managed the transmission blocking stage experiments at Imperial College
David M. Dranow	Beryllium Discovery Corp.	Crystallography: resolution of *Cp*KRS- inhibitor complexes, reviewed manuscript
Karen Dowers	University of Dundee	Development of biochemical assays
James Duffy	Medicines for Malaria Venture	Project coordination
Thomas E. Edwards	Beryllium Discovery Corp	Project supervision, reviewing structures, and scientific oversight
Alan H. Fairlamb	University of Dundee	Data analysis, interpretation and design of mode of inhibition experiments, reviewed manuscript
Liam Ferguson	University of Dundee	Pharmacokinetic studies
David Floyd	Structure-guided Drug Discovery Coalition	Project mentor, scientific advisor
Barbara Forte	University of Dundee	*In silico* drug design, interpretation of biological activity data, *in vitro* DMPK and *in vivo* PK and efficacy data, iterative drug design and chemical synthesis
Francisco Javier Gamo	DDW, GSK	Designed, conducted, and analyzed data for *in vitro* evaluation of *P. falciparum* parasite reduction rate

Ian H. Gilbert	University of Dundee	Supervised the drug discovery programme, led the writing of the manuscript, led and planned project
David W. Gray	University of Dundee	Supervised the *P. falciparum* parasite screening and biochemical assays
Irene Hallyburton	University of Dundee	Designed, conducted, and analyzed data for *in vitro* evaluation *Plasmodium falciparum* blood stage activity
Benjamin G. Horst	University of Washington	Conducted the small molecule screens that uncovered the hit chromome molecule
Matthew A. Hulverson	University of Washington	Conducted and anayzed data for the in vitro evaluation of *Cryptosporidium parvum* activity
Christopher D. Huston	University of Vermont	Supervised the *C. parvum* screening, time-kill experiments and *C. parvum* efficacy studies
Chimed Jansen	University of Dundee	*in silico* drug design, computational chemistry
María Belén Jiménez-Díaz	TAD	Design, execution, and analysis of in vivo efficacy study in the *P. falciparum* mouse model.
Rajiv S. Jumani	University of Vermont	Designed, conducted, and analyzed data for in vitro evaluation of *C. parvum* activity and time-kill experiments
Dennis E. Kyle	University of South Florida	Designed, conducted, and analyzed data for in vitro evaluation of *P. vivax* liver stage activity
Donald D. Lorimer	Beryllium Discovery Corp	Project supervision, review of structural data and implications, and scientific oversight
Melissa S. Love	CALIBR	Carried out the assays against different strains of *C. parvum* and *C. hominis*
Steven Maher	University of Georgia	*P. vivax* liver schizont and hypnozite assays
Pascal Mäser	SwissTPH	Contributed to writing the paper
Holly Matthews	Imperial College	Carried out the transmission blocking assays at Imperial College
Case W.McNamarra	CALIBR	Contributed to the design of the panel of *Cryptosporidium* isolates.
Peter Miller	University of Vermont	Conducted anti-*Cryptosporidium* NSG mouse efficacy studies
David Matthews	Structure-guided Drug Discovery Coalition	Project mentor, scientific advisor
Peter J. Myler	CIDR Center for Infectious Disease Research	Assisted in project design and directed SSGCID cloning, expression and crystallization efforts
Stephen Nakazawa Hewitt	University of Washington	Conducted screening campaign of TCAMS library with luciferase ATP consumption test
Neil R. Norcross	University of Dundee	*in silico* drug design, interpretation of biological activity data, *in vitro* DMPK and *in vivo* PK and efficacy data, iterative drug design
Sandra O'Neil	University of Dundee	Optimisation of Kinase Glo *Pf*KRS1 assay
Kayode K. Ojo	University of Washington	Conducted luciferase ATP consumption test based *Cp, Hs* KRS biochemical assays
Maria Osuna-Cabello	University of Dundee	DMPK bioanalysis

Caroline Peet	University of Dundee	Interpretation of biological activity data, *in vitro* DMPK and *in vivo* PK and efficacy data, co-ordination of in vitro DMPK activities
Andrei V. Pisliakov	University of Dundee	Supervision of molecular dynamics (MD) simulations and writing paper
Erika Pinto	University of Dundee	Pharmacokinetic studies
João Pedro Pisco	University of Dundee	Designed, conducted and analyzed data for the mode of inhibition studies and K_m determination
John Post	University of Dundee	Biochemical assay screening
Kevin Read	University of Dundee	Supervised the drug discovery programme, co-led project
Jennifer Riley	University of Dundee	Conducted *in vitro* DMPK studies on compounds
David Robinson	University of Dundee	Crystallography: resolution of *Cp*KRS and *Pf*KRS1- inhibitor complexes. Contributed to writing of paper
Matthias Rottmann	SwissTPH	Designed, collected and analyzed data for the in vitro activity testing with resistant strains K1 and TM90C2B and contributed to writing the manuscript
Laura Maria Sanz	DDW, GSK	The parasite reduction rate assay
Paul Scullion	University of Dundee	Designed, conducted and analyzed data for the metabolite identification studies
Frederick R. C. Simeons	University of Dundee	Pharmacokinetic studies
Robert Sinden	Imperial College	Coordinated work on male/ female gamete formation assays.
Amit Sharma	International Centre for Genetic Engineering and Biotechnology, New Delhi	Data collection and structure determination of *Pf*KRS1-drug complexes. Supervision of crystallography: resolution of *Pf*KRS1-inhibitor complexes
Arvind Sharma	International Centre for Genetic Engineering and Biotechnology, New Delhi	Crystallised and solved *Pf*KRS1-complex
Sharon M. Shepherd	University of Dundee	Conducted protein expression and purification for *Pf*, *Cp* and *Hs*KRS
Yoko Shishikura	University of Dundee	Conducted In vitro DMPK studies on compounds
Erin E. Stebbins	University of Vermont	Conducted in vitro anti-*Cryptosporidium* dose-response and time-kill curve studies.
Laste Stojanovski	University of Dundee	Pharmacokinetic studies
Ursula Straschil	Imperial College	
Boris Striepen	University of Pennsylvania	Supervision of *C. parvum in vivo* efficacy studies
Fabio K. Tamaki	University of Dundee	Development of biochemical assays
Jevgenia Tamar	University of Dundee	Crystallography: resolution of *Pf*KRS1- inhibitor complexes
Leah S. Torrie	University of Dundee	Design and supervision of mode of inhibition and Km determination, reviewed manuscript

Amélie Vantaux	Institut Pasteur du Cambodge	Obtained *P. vivax* sporozoites for liver stage assays
Wesley C. Van Voorhis	University of Washington	Supervision of *Cp*, *Hs* KRS biochemical assays, library screening, *C. parvum* in vitro assay and thermal shifts. Helped to write manuscript.
Sumiti Vinayak	University of Georgia	Design, execution and analysis of *in vivo* efficacy of compound **5** in the Nluc cryptosporidiosis INF-γ -knockout mouse model, and reviewed manuscript.
Chris Walpole	SDDC-SGC Toronto	SDDC project director, compound design, reviewed manuscript
Elizabeth A. Winzeler	University of California, San Diego	Design and support of hepatic stage assays
Benoît Witkowski	Institut Pasteur du Cambodge	Obtained *P. vivax* sporozoites for liver stage assays
Sergio Wittlin	Swiss TPH	Designed, collected and analyzed data from SCID mouse experiments and contributed to writing the article
Paul G. Wyatt	Dundee	Assisted in project design and management.
Manickam Yogavel	International Centre for Genetic Engineering and Biotechnology, New Delhilhi	Crystallised and solved *Pf*KRS1-drug complexes
Fabio Zuccotto	University of Dundee	Computational chemistry modelling

References

1. Choi R*, et al.* (2011) Immobilized metal-affinity chromatography protein-recovery screening is predictive of crystallographic structure success. *Acta Crystallogr Sect F Struct Biol Cryst Commun* 67:998-1005.
2. Bryan CM*, et al.* (2011) High-throughput protein production and purification at the Seattle Structural Genomics Center for Infectious Disease. *Acta Crystallogr Sect F Struct Biol Cryst Commun* 67:1010-1014.
3. Gamo FJ*, et al.* (2010) Thousands of chemical starting points for antimalarial lead identification. *Nature* 465:305-310.
4. Mersch-Sundermann V, Knasmuller S, Wu XJ, Darroudi F, & Kassie F (2004) Use of a human-derived liver cell line for the detection of cytoprotective, antigenotoxic and cogenotoxic agents. *Toxicology* 198:329-340.
5. Trager W & Jensen JB (1976) Human malaria parasites in continuous culture. *Science* 193:673-675.
6. Dorn A, Stoffel R, Matile H, Bubendorf A, & Ridley RG (1995) Malarial haemozoin/beta-haematin supports haem polymerization in the absence of protein. *Nature* 374:269-271.
7. Huber W & Koella JC (1993) A comparison of three methods of estimating EC50 in studies of drug resistance of malaria parasites. *Acta Trop* 55:257-261.
8. Sanz LM*, et al.* (2012) *P. falciparum* in vitro killing rates allow to discriminate between different antimalarial mode-of-action. *Plos One* 7:e30949.
9. Bessoff K, Sateriale A, Lee KK, & Huston CD (2013) Drug repurposing screen reveals FDA-approved inhibitors of human HMG-CoA reductase and isoprenoid synthesis that block Cryptosporidium parvum growth. *Antimicrob Agents Chemother* 57:1804-1814.
10. Jumani RS*, et al.* (2018) A Novel Piperazine-Based Drug Lead for Cryptosporidiosis from the Medicines for Malaria Venture Open-Access Malaria Box. *Antimicrob Agents Chemother* 62:e01505-01517.
11. Roth A*, et al.* (2018) A comprehensive model for assessment of liver stage therapies targeting *Plasmodium vivax* and *Plasmodium falciparum*. *Nat Commun* 9:1837.
12. Swann J*, et al.* (2016) High-Throughput Luciferase-Based Assay for the Discovery of Therapeutics That Prevent Malaria. *ACS Infect. Dis.* 2:281-293.
13. Plouffe DM*, et al.* (2016) High-Throughput Assay and Discovery of Small Molecules that Interrupt Malaria Transmission. *Cell Host Microbe* 19:114-126.
14. Delves MJ*, et al.* (2016) Routine in vitro culture of *P. falciparum* gametocytes to evaluate novel transmission-blocking interventions. *Nat Protoc* 11:1668-1680.
15. Patterson S*, et al.* (2013) The R enantiomer of the antitubercular drug PA-824 as a potential oral treatment for visceral Leishmaniasis. *Antimicrob. Agents Chemother.* 57:4699-4706.
16. Fagerberg JH*, et al.* (2010) Dissolution Rate and Apparent Solubility of Poorly Soluble Drugs in Biorelevant Dissolution Media. *Mol. Pharmaceutics* 7:1419-1430.
17. Valko K, Bevan C, & Reynolds D (1997) Chromatographic Hydrophobicity Index by Fast-Gradient RP-HPLC: A High-Throughput Alternative to log P/log D. *Anal Chem* 69:2022-2029.
18. Sokolova AY*, et al.* (2010) Cross-resistance to nitro drugs and implications for treatment of human African trypanosomiasis. *Antimicrob. Agents Chemother.* 54:2893-2900.
19. Jimenez-Diaz MB*, et al.* (2009) Improved murine model of malaria using *Plasmodium falciparum* competent strains and non-myelodepleted NOD-scid IL2Rgammanull mice engrafted with human erythrocytes. *Antimicrob. Agents Chemother.* 53:4533-4536.
20. Angulo-Barturen I*, et al.* (2008) A murine model of *falciparum*-malaria by *in vivo* selection of competent strains in non-myelodepleted mice engrafted with human erythrocytes. *Plos One* 3:e2252.

21. Jimenez-Diaz MB, *et al.* (2009) Quantitative measurement of *Plasmodium*-infected erythrocytes in murine models of malaria by flow cytometry using bidimensional assessment of SYTO-16 fluorescence. *Cytometry A* 75:225-235.
22. Manjunatha UH, *et al.* (2017) A Cryptosporidium PI(4)K inhibitor is a drug candidate for cryptosporidiosis. *Nature* 546:376-380.
23. Vinayak S, *et al.* (2015) Genetic modification of the diarrhoeal pathogen *Cryptosporidium parvum*. *Nature* 523:477-480.
24. Fang P, *et al.* (2015) Structural Basis for Specific Inhibition of tRNA Synthetase by an ATP Competitive Inhibitor. *Chem Biol* 22:734-744.
25. Case DA, *et al.* (2016) AMBER 2016, University of California, San Francisco.
26. Maier JA, *et al.* (2015) ff14SB: Improving the Accuracy of Protein Side Chain and Backbone Parameters from ff99SB. *J Chem Theory Comput* 11:3696-3713.
27. Frisch MJ, *et al.* (2004) Gaussian 03, Revision C.02, Gaussian, Inc., Wallingford CT.
28. Cornell WD, Cieplak P, Bayly CI, & Kollman PA (1993) Application of RESP charges to calculate conformational energies, hydrogen-bond energies, and free-energies of solvation. *J. Am. Chem. Soc.* 115:9620-9631.
29. Horn AH (2014) A consistent force field parameter set for zwitterionic amino acid residues. *J Mol Model* 20:2478.
30. Darden T, York D, & Pedersen L (1993) Particle Mesh Ewald - An N.Log(N) method for Ewald sums in large systems. *J. Chem. Phys.* 98:10089-10092.
31. Ryckaert JP, Ciccotti G, & Berendsen HJC (1977) Numerical-integration of cartesian equations of motion of a system with constraints - molecular-dynamics of n-alkanes. *J. Comput. Phys.* 23:327-341.
32. Delano WL (2002) The PyMOL Molecular Graphics System, DeLano Scientific: San Carols, CA.
33. Humphrey W, Dalke A, & Schulten K (1996) VMD: visual molecular dynamics. *J Mol Graph* 14:33-38, 27-28.
34. (2017) Maestro version 11.1. Schrodinger, LLC, New York.
35. Miller BR, 3rd, *et al.* (2012) MMPBSA.py: An Efficient Program for End-State Free Energy Calculations. *J Chem Theory Comput* 8:3314-3321.
36. Khan S, Sharma A, Belrhali H, Yogavel M, & Sharma A (2014) Structural basis of malaria parasite lysyl-tRNA synthetase inhibition by cladosporin. *J Struct Funct Genomics* 15:63-71.
37. Otwinowski Z & Minor W (1997) [20] Processing of X-ray diffraction data collected in oscillation mode. *Methods Enzymol* 276:307-326.
38. McCoy AJ, *et al.* (2007) Phaser crystallographic software. *J Appl Crystallogr* 40:658-674.
39. Adams PD, *et al.* (2010) PHENIX: a comprehensive Python-based system for macromolecular structure solution. *Acta crystallographica. Section D, Biological crystallography* 66:213-221.
40. Kabsch W (2010) Integration, scaling, space-group assignment and post-refinement. *Acta Crystallogr D Biol Crystallogr* 66:133-144.
41. Evans PR & Murshudov GN (2013) How good are my data and what is the resolution? *Acta Crystallogr D Biol Crystallogr* 69:1204-1214.
42. Murshudov GN, *et al.* (2011) REFMAC5 for the refinement of macromolecular crystal structures. *Acta Crystallogr D Biol Crystallogr* 67:355-367.
43. (1994) The CCP4 suite: programs for protein crystallography. *Acta Crystallogr D Biol Crystallogr* 50:760-763.
44. Emsley P & Cowtan K (2004) Coot: model-building tools for molecular graphics. *Acta Crystallogr D Biol Crystallogr* 60:2126-2132.
45. Winter G, Lobley CM, & Prince SM (2013) Decision making in xia2. *Acta crystallographica. Section D, Biological crystallography* 69:1260-1273.
46. McCoy AJ (2007) Solving structures of protein complexes by molecular replacement with Phaser. *Acta Crystallogr D Biol Crystallogr* 63:32-41.
47. Chen VB, *et al.* (2010) MolProbity: all-atom structure validation for macromolecular crystallography. *Acta Crystallogr D Biol Crystallogr* 66:12-21.
